# Bacteriophages in the environmental control of antimicrobial resistance: opportunities, risks, and surveillance challenges

**DOI:** 10.3389/fmicb.2026.1938962

**Published:** 2026-09-25

**Authors:** Imane El Belghiti, Maryem Wardi, Abdulmumini Baba Amin, Zohra Lemkhente, Khalid Sadki, Fatima Boubrik, Ahmed Belmouden

**Affiliations:** 1 Laboratory of Cellular Biology and Molecular Genetics, Faculty of Sciences, Ibn Zohr University, Agadir, Morocco; 2 Laboratory for Gastrointestinal Microbiology, Jiangsu Key Laboratory of Gastrointestinal Nutrition and Animal Health, National Centre for International Research on Animal Gut Nutrition, College of Animal Science and Technology, Nanjing Agricultural University, Nanjing, China; 3 Department of Animal Science, Federal University Dutse, Dutse, Jigawa, Nigeria; 4 Laboratory of Medical-Surgical, Biomedicine and Infectiology Research, Faculty of Medicine and Pharmacy, Ibn Zohr University, Agadir, Morocco; 5 Research Laboratory in Oral Biology and Biotechnology, Faculty of Dental Medicine, Mohammed V University in Rabat, Rabat, Morocco

**Keywords:** antibiotic resistance genes, antimicrobial resistance, bacteriophages, environmental resistome, One Health, phage-based biocontrol, surveillance

## Abstract

Antimicrobial resistance (AMR) is a major global public health challenge, driven by the persistence and dissemination of antibiotic-resistant bacteria and antibiotic resistance genes (ARGs). These resistance determinants can accumulate and spread across diverse environmental compartments, including wastewater, hospital effluents, agricultural systems, and landfills, facilitating their dissemination through mechanisms such as horizontal gene transfer. Bacteriophages have emerged as promising tools for the targeted control of antibiotic-resistant bacterial populations, as their lytic activity can overcome conventional antibiotic resistance mechanisms and effectively target multidrug-resistant (MDR) and extensively drug-resistant (XDR) bacteria. In addition, phage-encoded enzymes, including endolysins and polysaccharide depolymerases, can disrupt bacterial cell structures and biofilms, further enhancing their potential as alternative antimicrobial strategies. However, the role of bacteriophages in AMR dynamics is inherently dual, as lytic phages can reduce antibiotic-resistant bacterial populations, whereas temperate phages may contribute to the dissemination of ARGs through transduction-mediated gene transfer. This duality represents an important biosafety concern that requires rigorous assessment before their large-scale application in environmental settings. This review critically examines the role of bacteriophages in addressing environmental antimicrobial resistance within a One Health framework. It first explores environmental ARG reservoirs and current surveillance approaches, followed by bacteriophage biology and mechanisms of action, their applications in clinical medicine, veterinary science, and environmental biocontrol, and finally the biological constraints and strategies to address these challenges. This work integrates environmental surveillance with phage-based intervention strategies to support the rational and responsible deployment of bacteriophages as complementary tools in the global response to AMR.

## 1 Introduction

Antimicrobial resistance (AMR) is a major global public health threat, contributing to nearly 5 million deaths worldwide (World Health Organization, 2022; Naghavi et al., 2024). While the misuse and overuse of antibiotics remain major drivers of antimicrobial resistance, increasing evidence highlights the important role of environmental pathways in its emergence and dissemination. Anthropogenic activities, including pharmaceutical manufacturing, agricultural practices, and healthcare operations, release antibiotic residues and resistance determinants into the environment through wastewater, agricultural runoff, and hospital effluents, creating conditions that can promote their persistence and spread. As a result, antibiotic resistance genes (ARGs) build up in environmental matrices like soils, sediments, and aqueous systems (Berendonk et al., 2015). Studies on ARGs found very high concentrations of these resistance elements, with up to 10^6^–10^10^ copies per gram of soil/sediment and 10^4^–10^8^ per milliliter of wastewater, indicating environmental routes as one of the major reservoirs of resistance determinants (He et al., 2020).

Once introduced into the environment, ARGs remain persistent in various ecological niches and get distributed by horizontal gene transfer (HGT), resulting in the emergence of multidrug-resistant (MDR) and extensively drug-resistant (XDR) strains (Zhai et al., 2025). These dynamics are further amplified by the co-selection pressure from residual antibiotics, heavy metals, and disinfectants in environmental matrices, enriching resistant bacteria continuously even in the absence of antibiotics (Bekele et al., 2026).

Despite of all this awareness, there are still significant gaps in the methodology of environmental surveillance of ARGs and integration of the information into the AMR strategies. All existing methods of ARGs reduction, such as the improvements in the quality of wastewater treatment facilities, the adoption of the appropriate measures of the antibiotic stewardship programs and agricultural best practices can reduce but not completely eliminate the environmental burden of resistance. The constant emissions of wastewater containing ARGs, mobile genetic elements (MGEs), and viable resistant bacteria necessitate the implementation of additional strategies of intervention targeting the direct reduction of resistant bacterial populations (Manoharan et al., 2022; Wardi et al., 2024a).

Addressing these challenges, it is necessary to implement the integrated strategy of mitigation including responsible use of antibiotics, improvement of wastewater treatment technologies and continuous monitoring of antibiotic-resistant bacteria and ARGs in the environment. In this situation, bacteriophages - viruses targeting bacteria have become very promising biological agents for the biocontrol of antibiotic-resistant bacteria due to their high host specificity, self-reproduction ability, and independence from the mechanisms of conventional antibiotic resistance (Pirnay et al., 2024). However, the role of bacteriophages in environmental dynamics of AMR is ambiguous since while lytic bacteriophages help to reduce the population of resistant bacteria through their targeted infection, temperate bacteriophages can increase the spread of ARGs through horizontal gene transfer (Colavecchio et al., 2017; González-Villalobos et al., 2025).

Substantial progress has been made in understanding environmental antimicrobial resistance (AMR) and bacteriophage biology, yet these two fields have rarely been considered together within a unified framework. Previous reviews have generally focused either on environmental resistome surveillance (Zhang et al., 2025) or on phage applications in clinical settings, mapping the drug-development pipeline from phage bank to bedside and identifying gaps in cocktail design and clinical trial standardization (Kim et al., 2025), with limited attention to their broader environmental and ecological dimensions.

This review addresses this gap by providing an integrated overview of environmental ARG reservoirs and their surveillance, bacteriophage properties and mechanisms of action, and their applications in clinical and non-clinical settings, including environmental biocontrol. It further examines emerging phage-based strategies, ecological implications, and key biosafety and regulatory considerations, with particular emphasis on the dual role of phages as antimicrobial agents and potential vectors of ARG dissemination. These aspects are considered within an integrated One Health framework to support the safe and rational development of phage-based strategies against AMR.

## 2 Environmental reservoirs and dissemination of antimicrobial resistance genes

Antimicrobial resistance genes refer to specific genetic elements which allow microorganisms, especially bacteria, to grow and survive even when antimicrobials are present in their environment. ARGs can confer several modes of resistance including degradative activity on antibiotics, modifications of binding sites of antibiotics, decreased membrane permeability and antibiotic efflux activity. These genes have increasingly been recognized to be an important public health issue owing to their involvement in antibiotic resistance problem.

### 2.1 Mechanisms of antibiotic resistance and dissemination of ARGs

Antibiotic resistance is a natural evolutionary phenomenon that predates the clinical use of antibiotics. Many antibiotics are derived from compounds naturally produced by bacteria and fungi as part of microbial competition, creating selective pressures that have shaped resistance mechanisms in microbial communities over evolutionary time. Consequently, some microorganisms possess intrinsic resistance mechanisms, while others can acquire antibiotic resistance genes (Kong et al., 2020).

The dissemination of ARGs occurs through two main processes: vertical transmission which refers to the inheritance of resistance genes from parent cells to their progeny during bacterial replication and horizontal gene transfer which enables the exchange of genetic material between bacteria of the same generation, greatly accelerating the spread of resistance determinants across microbial communities (Von Wintersdorff et al., 2016).

Horizontal gene transfer can be achieved via conjugation, transformation, and transduction. The first, involves the transfer of genetic material from one bacterium to another through direct contact of cells. This method is responsible for the transfer of genes encoding for drug resistance. The second, transformation refers to the uptake of extracellular DNA released from lysed cells by competent bacteria. Transduction is facilitated by bacteriophages, which may accidentally package fragments of bacterial DNA, including antibiotic resistance genes, and transfer them to new bacterial hosts during subsequent infection cycles (Tokuda and Shintani, 2024) (Figure 1A).

**Figure 1:**
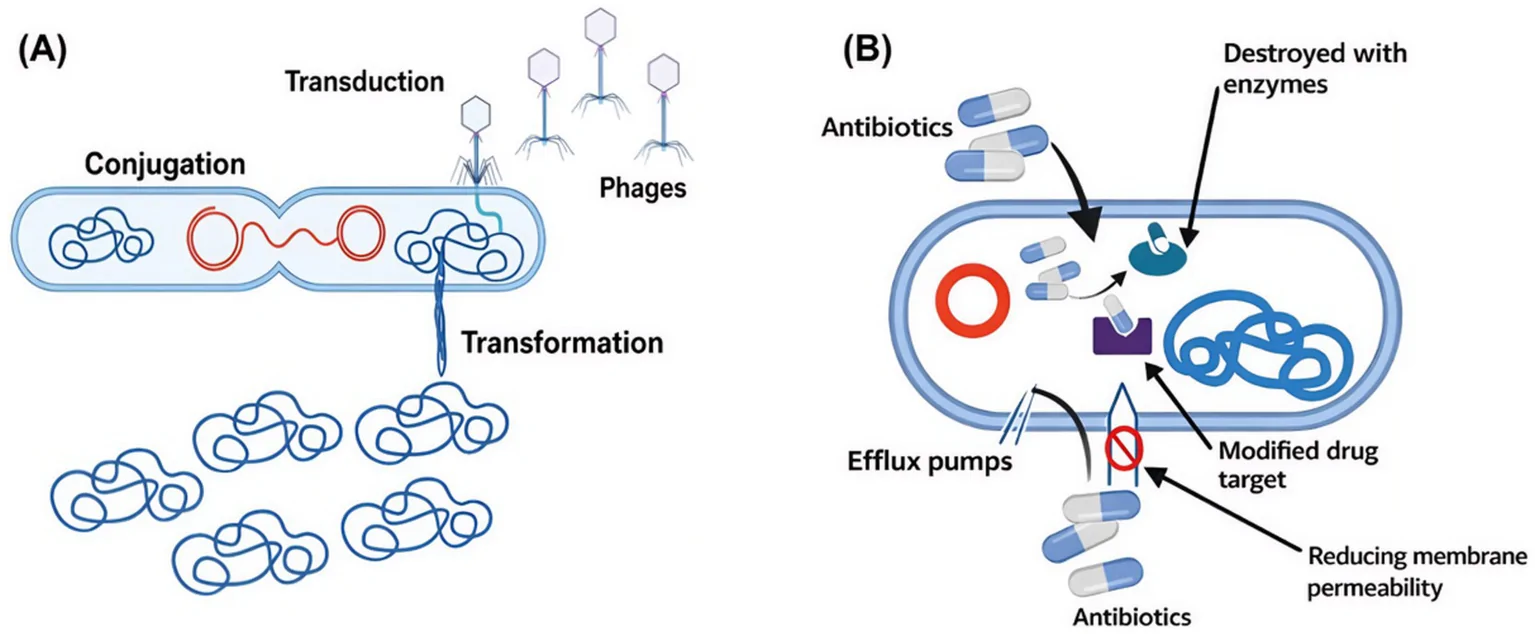
Major mechanisms of gene dissemination and antibiotic resistance. **(A)** Horizontal gene transfer mechanisms including conjugation, transformation, and phage-mediated transduction. **(B)** Major biochemical resistance mechanisms such as enzymatic inactivation, reduced permeability, target modification, and active efflux (Created by the authors).

Generalized and specialized transduction are the two major types of transduction processes. The generalized type of transduction usually takes place in the lytic phase of certain bacteriophages, where accidental packaging of random fragments of the host’s genomic DNA into bacteriophage particles leads to inclusion of such sequences as ARGs and other MGEs. Specialized transduction involves integration of the bacteriophage genome into the bacterial genome in the form of prophages. During prophage excision, neighboring bacterial genes can be co-packaged with phage DNA and subsequently transferred to new host cells (Borodovich et al., 2022). Although specialized transduction generally mobilizes a more limited set of genes, it may still contribute to the spread of resistance determinants within bacterial populations (González-Villalobos et al., 2025).

Indeed, the acquisition of resistance genes through horizontal gene transfer enables bacteria to develop a wide range of adaptive mechanisms that limit the efficacy of antimicrobial agents. Among the most prevalent resistance mechanisms, four main modes stand out (Bush and Bradford, 2016) (Figure 1B):

- Enzymatic inactivation: where bacteria produce enzymes capable of degrading or modifying antibiotics, such as β-lactamases that hydrolyze β-lactam antibiotics.
- Reduced membrane permeability: resulting from alterations in membrane porins that limit antibiotic entry into bacterial cells.
- Modification of antibiotic targets: which reduces the binding affinity of antimicrobial agents for their cellular targets.
- Active efflux: where specialized transport systems expel antibiotics from the bacterial cell before they can exert their antimicrobial effect.

Despite these observations, the overall quantitative contribution of phage-mediated ARG transfer in natural environments remains debated. Several studies indicate that transduction occurs less frequently than plasmid-mediated conjugation, suggesting that its overall impact may be comparatively limited (Davies, 1996).

These findings highlight the dual role of bacteriophages in AMR dynamics as potential agents for bacterial control on one hand, and as possible vectors of ARG dissemination on the other a balance that is discussed in detail in Section 3.2.

### 2.2 Environment as a reservoir of ARGs

Environmental compartments act as dynamic reservoirs in which antibiotics, resistant bacteria, and resistance genes may accumulate, persist, and circulate across interconnected human, animal, and ecological systems.

Within these reservoirs, frequent genetic exchanges and the high plasticity of microbial genomes facilitate the spread of resistance determinants between bacterial species. The emergence of multidrug-resistant and pan-resistant bacterial strains, together with the dissemination of resistance genes across environmental, animal, and human reservoirs, may be facilitated by these processes.

In this review, we present the main sources of environmental antibiotic resistance pollution (Figure 2).

**Figure 2:**
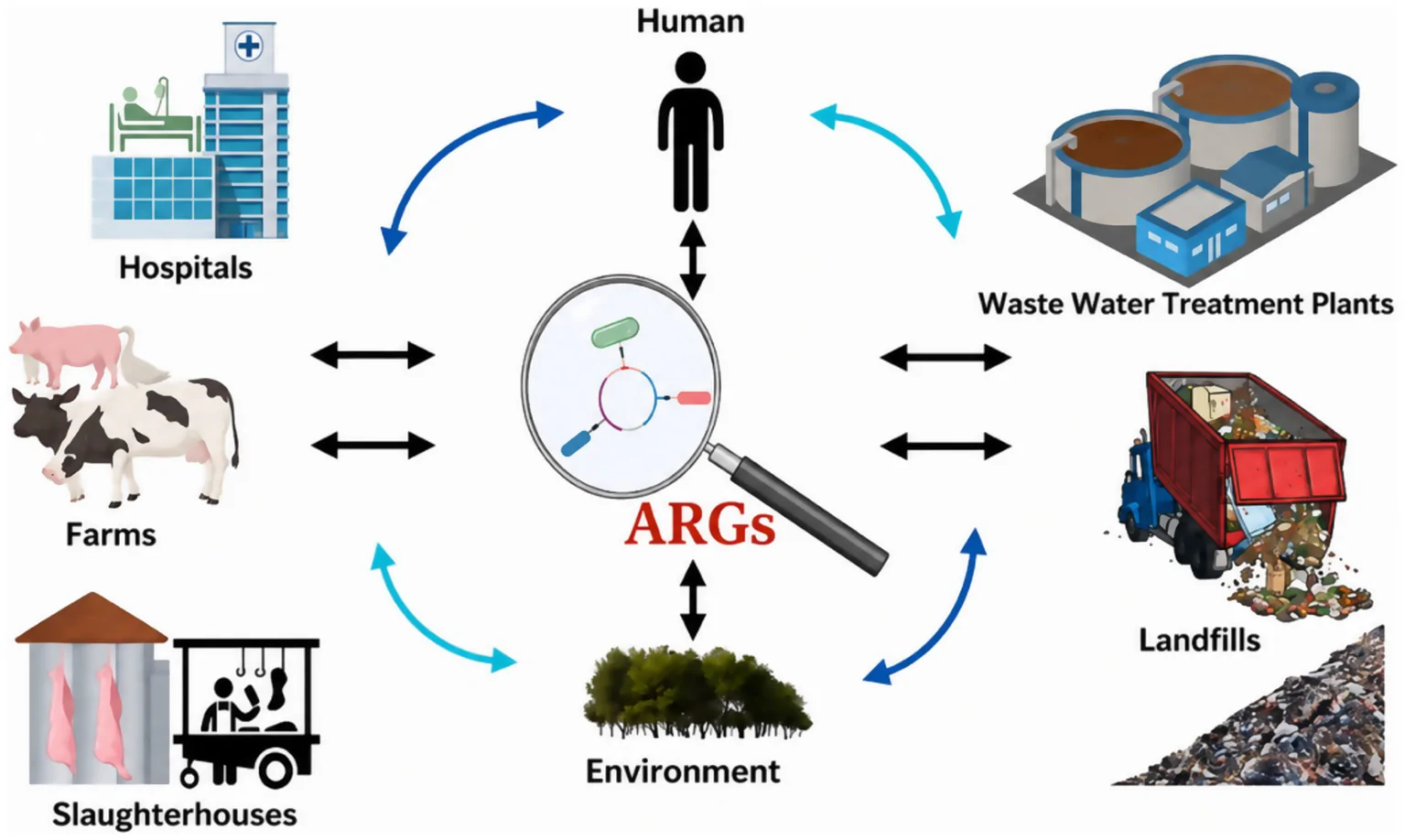
Major environmental sources of dissemination of ARGs. Human activities, including the release of effluents from hospitals, livestock farming, discharge of slaughterhouses, Wastewater Treatment Plants (WWTP), and landfill leachates are always introducing ARGs and antibiotic-resistant bacteria to the environment. Bidirectional arrows indicate the reciprocal exchange and circulation of ARGs among humans, environmental reservoirs, and anthropogenic sources. Blue arrows highlight the role of environmental compartments, particularly WWTPs and other contaminated environmental reservoirs, as secondary dissemination pathways facilitating the transmission of ARGs back to the human population (Created by the authors).

#### 2.2.1 Wastewater

WWTPs receive large volumes of wastewater from domestic, hospitals, and industries. This wastewater constitutes an important reservoir of antibiotic resistance genes, which can be exchanged among bacteria through horizontal gene transfer. Consequently, the discharge of untreated wastewater into the environment represents a significant risk to both human health and ecosystem integrity.

Advanced techniques such as Next-Generation Sequencing (NGS), quantitative PCR (qPCR), and digital droplet PCR (ddPCR) now allow detailed characterization of resistance genes in wastewater, making it a key focus for monitoring antibiotic resistance (Ekwanzala et al., 2020; Jankowski et al., 2022; Wardi et al., 2024a,b). A summary of the major environmental matrices, recurrent ARG markers, mobility elements, surveillance approaches, risk drivers, and control levers reported in wastewater treatment plants is provided in Table 1, while an extended dataset of ARGs detected in wastewater treatment plants is presented in Supplementary Table S1.

**Table 1:** Comprehensive summary of ARGs across major environmental reservoirs: wastewater treatment plants, farms and slaughterhouses, hospitals, and landfills.

Key element	Wastewater (WWTP)	Farms and slaughterhouses	Hospitals	Landfills
Typical matrices	Influent/effluent, activated sludge, treatment units, receiving waters	Manure/slurry, farm water, sediments/aquaculture, dust/air, amended soils	Hospital effluents, sludge/sewage, receiving sediments, aerosols around hospitals	Leachates (young/aged), surrounding soils, ponds, site aerosols
Common ARG markers	β-lactamases (*bla_TEM_, bla_CTX-M_, bla_OXA_*, carbapenemases *bla_NDM/KPC/VIM_*), tetracyclines (*tetA/M/O/Q/W/X*), sulfonamides (*sul1/2/3*), macrolides (*ermB/ermC*), quinolones (*qnrA/B/S*), colistin (*mcr-1*)	Tetracyclines (*tetA/M/O/W/X*), sulfonamides (*sul1/2/3*), β-lactamases (*bla_TEM_, bla_CTX-M_*), quinolones (*qnrS/A*), macrolides (*ermB/ermA*), sometimes *mcr-1* / *NDM-1*	ESBL/β-lactamases (*bla_CTX-M_, bla_TEM_, bla_OXA_*), carbapenemases (*bla_NDM_, bla_KPC_, bla_OXA-48_, bla_VIM_*), vancomycin (*vanA*), + *sul/tet/erm* markers depending on site	Profiles dominated by *tet (tetO/W/M/Q…)*, *sul (sul1/sul2)*, *ermB*, sometimes *mexF*/transporters depending on studies
MGEs/mobility	intI1 (integrons), transposons (e.g., *Tn916*)	*intI1*, plasmid/transposon-associated genes	*intI1*, plasmid/transposon-associated genes	*intI1*, plasmid/transposon-associated genes
Surveillance methods	qPCR/HT-qPCR, Illumina metagenomics, ARG database pipelines	PCR/qPCR, metagenomics, isolate WGS	qPCR, metagenomics, WGS + typing (SRST2/ARG-Annot)	qPCR/ddPCR, Illumina metagenomics, 16S normalization
Risk drivers	High microbial load, co-selection, discharges to aquatic environments	Veterinary antibiotic use, animal density, manure management, runoff	Broad-spectrum antibiotic use, patient density, concentrated effluents	Waste heterogeneity, leachate age, climate/seasonality, groundwater infiltration
Control levers	Advanced treatments (ozonation/UF), source reduction, hospital pre-treatment	Antibiotic reduction, vaccination/hygiene, controlled composting, runoff barriers	Targeted pre-treatment/disinfection, integration in WWTP strategy, continuous surveillance	Leachate collection/treatment, containment, groundwater monitoring, reduction of antibiotic-containing waste
References	Guo et al. (2017), Freeman J. et al. (2018), Wardi et al. (2024a)	Latif et al. (2026)	Gupta et al. (2014), Inouye et al. (2014)	Wang et al. (2020)

ARGs: antimicrobial resistance genes; MGEs: mobile genetic elements; HT-qPCR: high-throughput quantitative PCR; UF: ultrafiltration; WGS: Whole Genome Sequencing; ESBL: extended-spectrum β-lactamases. Gene lists represent frequently reported markers and are not exhaustive. For the exhaustive list refer to Supplementary Table.

#### 2.2.2 Farms and slaughterhouses

Certain agricultural practices have promoted the emergence of resistant and multidrug-resistant bacteria, as well as the spread of antimicrobial resistance genes into the environment. This is because antibiotics are fed to the animals for increased production and treatment of infections among other reasons. This practice involves multiple classes of antibiotics, including tetracyclines (Granados-Chinchilla and Rodríguez, 2017), penicillins, and sulfonamides (Sarkar and Okafor, 2022). Soil amendment through animal excreta enriches the soil with resistant bacteria, which can be transmitted to humans through fresh vegetable produce and even contaminate groundwater. The table below (Table 1) summarizes environmental matrices, ARG marker genes, MGEs, monitoring methods, risk drivers and control strategies in farms and slaughterhouses, while a more detailed dataset of ARGs detected in these environments is provided in Supplementary Table S2.

#### 2.2.3 Hospitals

Healthcare institutions play a pivotal role in the dissemination of bacterial resistance (Meggersee and Abratt, 2015). The widespread use of broad-spectrum antibiotics in hospitals creates strong selective pressure, increasing the risk of transmission of resistant bacteria between patients. Since the 1940s, resistant strains of clinically important pathogens, including *Escherichia coli*, *Staphylococcus aureus*, and enterobacteria, have been increasingly reported and are now among the most frequently encountered pathogens in healthcare settings (Zaidah et al., 2017). These bacteria are responsible for severe nosocomial infections, leading to increased morbidity, mortality, and healthcare costs. Table 1 presents the main matrices, recurrent ARG markers, surveillance methods, risk drivers, and control strategies in hospital environments whereas an extended compilation of hospital-associated ARGs and detection methods is presented in Supplementary Table S3.

#### 2.2.4 Landfills

Leachate generated in landfills represents an additional environmental source of antibiotics, resistant bacteria, and antimicrobial resistance genes. This poses serious threats to human health and the environment, particularly groundwater, which can become contaminated through infiltration. On a global scale, leachates from landfills are stored in basins and typically undergo no treatment. Managing these effluents is a considerable challenge, especially since these wastes undergo biological evolution over time, potentially generating harmful and hazardous species. Table 1 summarizes the main matrices, recurrent ARG markers, surveillance approaches, risk drivers, and control measures in landfill environments, with a more comprehensive dataset detailed in Supplementary Table S4.

### 2.3 Surveillance of ARGs

In light of the growing recognition of the environment as a major reservoir and amplification site for ARGs, robust surveillance systems are urgently needed to monitor their occurrence, dissemination pathways, and ecological drivers.

Wastewater treatment plants (WWTPs) are recognized as critical hotspots for ARGs, where quantitative PCR (qPCR) and metagenomic approaches have been used to measure the abundance of genes such as (*bla_CTX-M_, ermB, tetO, sul1, qnrS*) highlighting their role in ARG persistence (Guo et al., 2017; Freeman C. N. et al., 2018).

Similarly, farms and slaughterhouses represent important reservoirs, where the whole-genome sequencing is increasingly used as a powerful surveillance tool in agricultural and food-production settings. Riaz et al. (2020) also used high-throughput sequencing to quantify the ARGs *intl1, intl2, bla_CTX-M_, sul1, oqxB, ere (A), tet (A), qnrD*. In recent years, mass spectrometry has replaced high-performance liquid chromatography (HPLC) for surveillance of the concentration of ATBs in organic waste on farms or even in slaughterhouses (Jamur et al., 2025).

Landfills are also widely recognized as a major source of antibiotics. Several ARGs have been found in landfill leachates, notably *bla (bla_CTXM_, bla_TEM_), sul (sul1, sul2), tet (tetO, tetQ, tetW)* and *ermB* (Wu et al., 2015; Wang et al., 2020), commonly identifier through qPCR and Illumina sequencing based on 16S rRNA (Liu et al., 2022).

In hospitals, a new method for rapid genomic surveillance in hospitals which is SRST2, named after an earlier tool SRST (Short Read Sequencing Typing), based on read mapping for rapid and accurate detection of genes, alleles and multilocus sequence types (MLST) from WGS data (Inouye et al., 2014). WGS can also be used and its results can be interrogated using BLAST or other search algorithms to identify genes or alleles of interest (Gupta et al., 2014).

### 2.4 Control strategies

The spread of antimicrobial resistance genes and antibiotic-resistant bacteria can be mitigated through interventions at three complementary levels (Allerberger et al., 2009; Goulas et al., 2018).

First, rationalizing antibiotic use is essential across human, animal, and agricultural promoting judicious prescribing practices, avoiding unnecessary treatments, and encouraging alternatives such as vaccines and preventive care help limit selective pressure (Binda et al., 2020).

Second, improving of waste management (including industrial, municipal, hospital, and agricultural waste). Effective treatment of liquid and solid wastes, such as advanced wastewater treatment and appropriate handling of medical and agricultural waste, can significantly reduce the dissemination of ARGs into the environment (Manoharan et al., 2022).

Finally, contaminated environments should be effectively managed because even low concentrations of antibiotics can select for multidrug-resistant bacteria, posing risks to human health, livestock, aquaculture, and agriculture. Policies such as the European Union Watch List for emerging pollutants highlight priority compounds, including amoxicillin and ciprofloxacin, and underscore the need for improving wastewater and sewage treatment technologies. Combining secondary and tertiary treatment processes enhances the removal efficiency of antibiotics and reduces environmental contamination (Bekele et al., 2026).

Overall, there are many ways in which the use of antibiotics can be rationalized and the levels of waste managed in order to minimize the occurrence and spread of antimicrobial resistance. Nonetheless, the rapid evolution of resistance often outpaces the development of new antibiotics. In this context, bacteriophage has recently become a topic of interest among researchers in search of novel means of treating resistant infections.

However, the effectiveness of these interventions depends not only on their technical feasibility but also on sustained governance, financial investment, and long-term implementation. Their impact may therefore remain limited where regulatory enforcement is weak, infrastructure investments are difficult to sustain, or environmental contamination is addressed only after it has become established. These limitations point to the need for complementary biological approaches, including bacteriophage-based biocontrol, which may offer more targeted strategies for controlling resistant bacterial populations and will be discussed in the following sections.

## 3 Bacteriophages: biology, mechanisms of action and dual role in AMR dynamics

### 3.1 Phage biology: structure and replication cycles

Bacteriophages are special viruses which specifically infect bacteria and can be found in abundance in nature, including water environment, soil and sewage treatment facilities (Principi et al., 2019). The development of molecular biology and electron microscopy made significant contributions to the knowledge about the structure and variety of bacteriophages. The structure of phage consists of nucleic acids which can be both single- and double-stranded DNA and RNA and are encased by protein capsid (Figure 3) (Hendrix et al., 1999).

**Figure 3:**
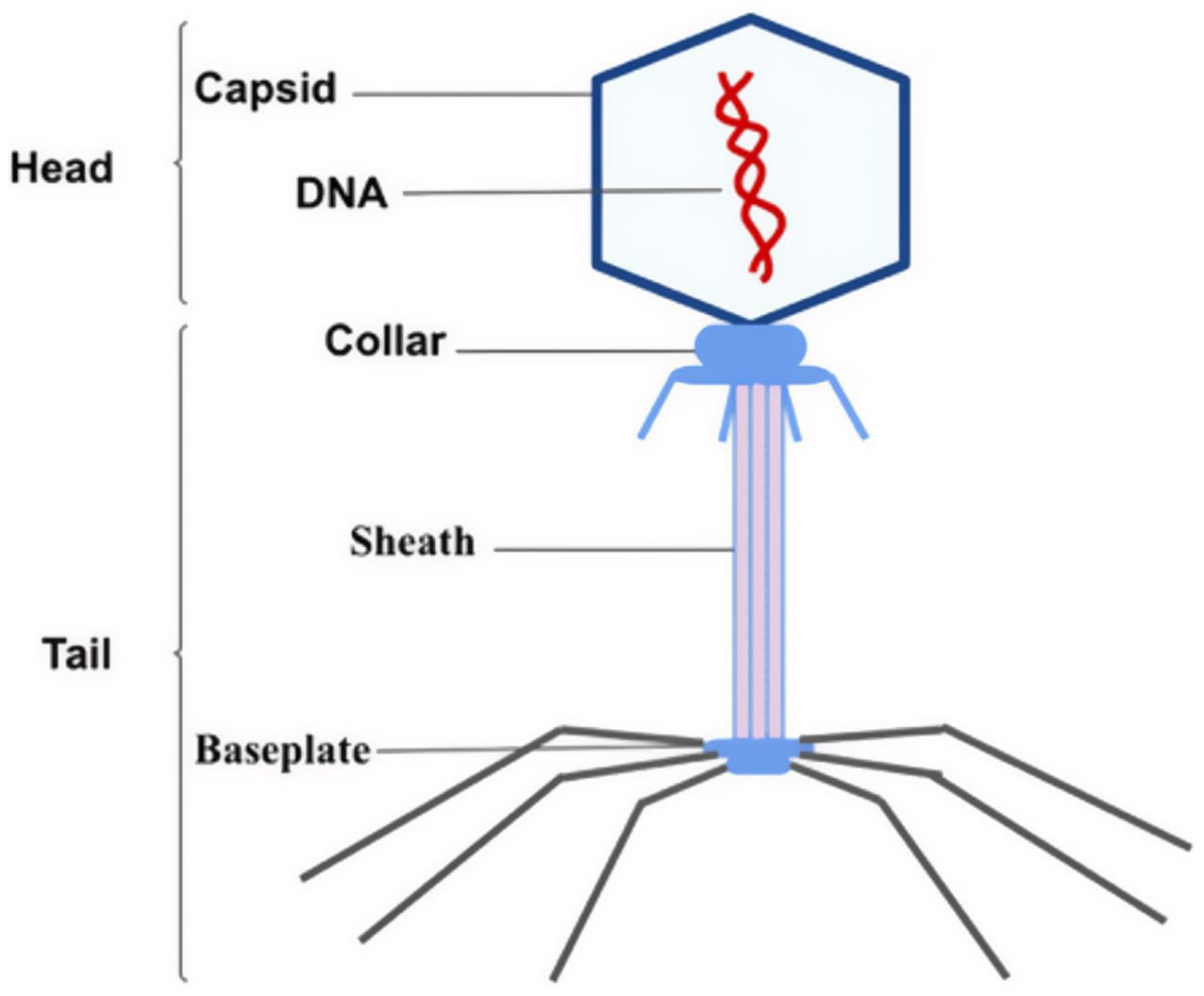
Structure of T4 bacteriophage (Created by the authors).

Importantly, bacteriophages lack cellular machinery that facilitates transcription, translation, and energy generation. In common with all other viruses, bacteriophages have no metabolism and depend on the biosynthetic capacity of the host cell to replicate (Clokie et al., 2011). Dependence on the host cell not only helps bacteriophages to multiply but also dictates the methods they use to infect bacterial cells. The lytic cycle and lysogenic cycle are the two main ways by which bacteriophages infect bacteria (Figure 4).

**Figure 4:**
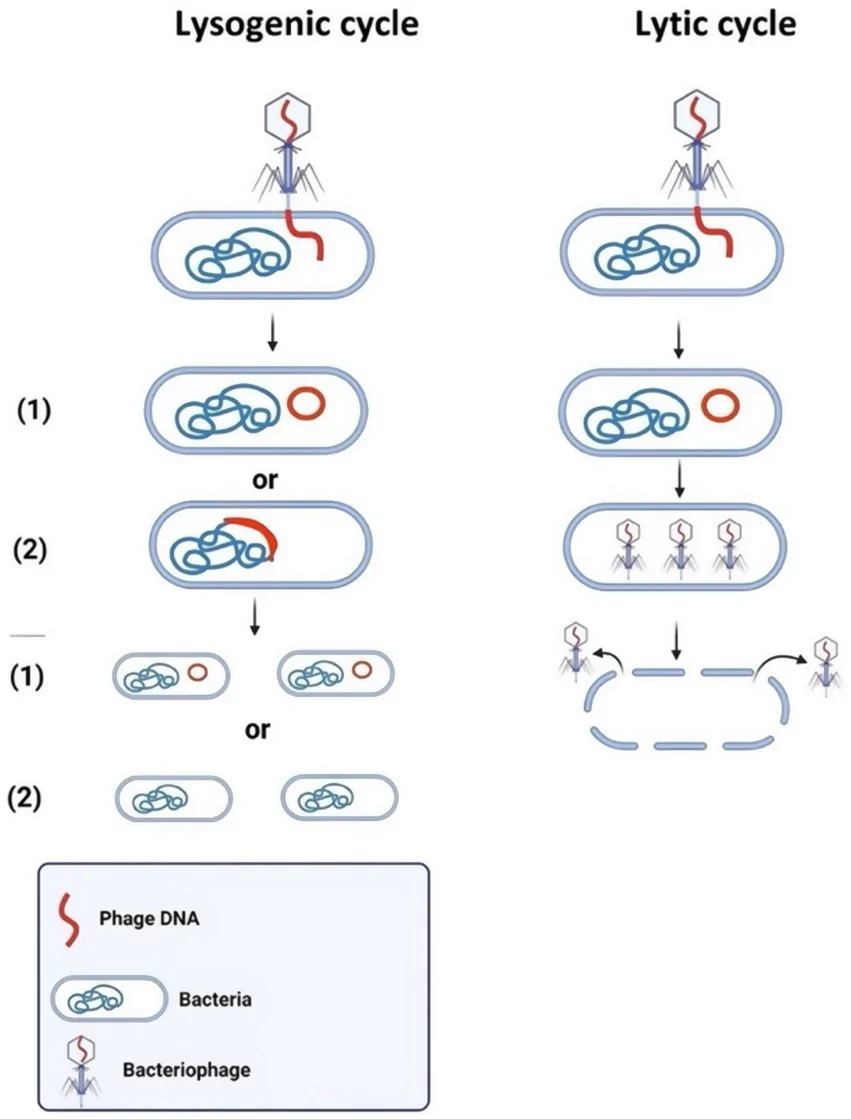
Lytic and lysogenic cycle (Created by the authors).

In the lytic cycle, the phage attaches to specific receptors on the bacterial surface and injects its genetic material into the host cell. The viral genome then directs the production of new phage components using the host cellular machinery. Newly assembled virions accumulate within the cell until the bacterial membrane is lysed, releasing numerous progeny phages into the surrounding environment (Abedon, 2011).

In contrast, the lysogenic cycle involves the integration of the phage genome into the bacterial chromosome as a prophage. In this state, the phage DNA replicates passively along with the host genome without causing immediate cell lysis. Under certain conditions, the prophage can be induced to exit the bacterial genome and enter the lytic cycle (Ferry et al., 2020).

Although lysogenic phages play an important role in bacterial evolution and horizontal gene transfer, lytic phages are generally preferred for therapeutic and biocontrol applications because they directly eliminate bacterial cells (Ansaldi et al., 2020).

### 3.2 Mechanisms of phage action against resistant bacteria

The antibacterial activity of bacteriophage against antibiotic resistance bacteria utilizes several mechanisms which are quite different from the traditional antibiotics. Understanding about such mechanisms is important in assessing the potential and limitations of using phages as a treatment strategy.

#### 3.2.1 Lytic activity and host cell destruction

Lytic phages are preferred for therapeutic applications because they eliminate bacteria through the lytic cycle (Section 3.1), resulting in bacterial cell lysis. Importantly, their antibacterial activity is independent of conventional antibiotic resistance mechanisms, allowing them to effectively kill multidrug-resistant bacteria (Loessner, 2005). This property represents a major advantage of phage therapy over conventional antibiotics for the treatment of multidrug-resistant infections.

#### 3.2.2 Endolysins and exolysins

Phage-encoded lytic enzymes, known as endolysins (or lysins), are produced at the end of the lytic cycle to degrade the bacterial peptidoglycan layer from within, triggering osmotic lysis and cell death. Endolysins have attracted considerable interest as standalone antibacterial agents, particularly against Gram-positive bacteria such as *Staphylococcus aureus*, vancomycin-resistant *Enterococcus* (VRE), and *Clostridioides difficile*, whose thick peptidoglycan layer is directly accessible from the outside (Loessner, 2005). When applied externally, endolysins can lyse bacteria rapidly within seconds to minutes without the need for phage replication, reducing the risk of bacterial resistance development to the enzyme itself. Several endolysins targeting MRSA, including SAL200 and SAL-1, have entered preclinical and clinical development (Fischetti, 2010). For Gram-negative bacteria, the outer membrane presents a barrier to endolysin penetration; however, engineered endolysins combined with outer-membrane-permeabilizing agents have shown promise against MDR *Acinetobacter baumannii* and *Pseudomonas aeruginosa* (Murray et al., 2021).

#### 3.2.3 Phage activity against biofilms

Biofilms structured communities of bacteria embedded in a self-produced extracellular matrix represent a particularly challenging target for antibiotic therapy, as the biofilm matrix acts as a physical barrier limiting antibiotic penetration and the metabolic dormancy of biofilm cells reduces their susceptibility to bactericidal agents. Bacteriophages have evolved specific strategies to penetrate and disrupt biofilms: many phages encode polysaccharide depolymerases that enzymatically degrade the exopolysaccharide components of the biofilm matrix, facilitating phage diffusion and access to bacterial cells within the biofilm (Pires et al., 2017). Studies have demonstrated that phages, particularly when used in cocktails or combined with antibiotics, can achieve significant reductions in biofilm biomass formed by *Pseudomonas aeruginosa*, *Staphylococcus aureus*, *Klebsiella pneumoniae*, and *E. coli* on both abiotic surfaces and in environmental matrices such as wastewater biofilms (Chegini et al., 2020). The synergistic combination of phages and antibiotics against biofilms is particularly noteworthy: phages can disrupt the biofilm structure and expose bacteria to antibiotics, while sub-inhibitory antibiotic concentrations may in some cases upregulate phage receptor expression, enhancing phage adsorption.

### 3.3 Utilization of bacteriophages as tools for environmental AMR control

As discussed in Section 2.1, the impact of phages on environment AMR is inherently twofold. On one hand, phages can spread AMR genes via transduction. On the other, they can be used as a tool to control antibiotic-resistant bacteria. Phages have been utilized in the environment to reduce bacterial populations in water treatment systems, hospital effluent, livestock, aquaculture, food processing systems, as well as in engineered systems where biofilms develop.

The use of phage cocktails is employed to enhance host range and slow down bacterial escape, although the exact composition of such cocktails depends on the particular environmental application (Viertel et al., 2014). If incorporated into environmental surveillance programs, source reduction, and traditional management approaches, phages could become an additional way to control the spread of AMR genes.

According to recent research, phages are effective in reducing bacterial concentrations in biofilms and wastewater matrices, which shows the potential of these organisms as natural and specific means to control environmental populations of resistant bacteria(Abedon et al., 2017; Chegini et al., 2020).

#### 3.3.1 Key opportunities and constraints of phage-based interventions

The efficiency of the usage of phage therapies depends greatly upon the target matrix, physicochemical parameters, the structure of microbial community, and the amount of contact of infective phage particles with bacteria hosts. It is especially significant for environmental situations, when wastewater, sludge, soil, biofilm, and water ecosystems are likely to affect the stability, diffusion, and accessibility of phages.

Bacteriophages represent an interesting alternative to the usual antibiotics, yet its use presupposes balancing benefits and limitations of phages. The killing action of phages differs from that of antibiotics by its ability to combat bacteria resistant to the latter and not affected by any existing mechanism of resistance (Viertel et al., 2014).

Moreover, phages have the ability to multiply inside bacteria, increasing the number of viruses and the efficiency of therapy (Dublanchet and Patey, 2011). High specificity towards certain strains of bacteria contributes to the preservation of microbial communities. Also, the use of phages decreases the environmental load caused by antibiotics, which supports ecologic balance.

Nevertheless, there are some limitations associated with phages. The usage of bacteriophages requires them to be strictly lytic since the presence of temperate phages increases the chance of the process of horizontal gene transfer as well as creation of bacteria population immune to phage attacks (Abedon et al., 2017). Phages and bacteria are involved in continuous co-evolution; thus, the bacterial system of protection (for example, restriction modification or CRISPR-Cas) can decrease the efficiency of phages in time.

This highlights the importance of selecting appropriate phages, performing comprehensive genomic analyses to ensure their safety and efficacy, and considering the use of phage cocktails to broaden the host range and reduce the emergence of phage-resistant bacteria (Abedon et al., 2017). However, the successful application of phages also depends on their stability in environmental matrices, as factors such as temperature, pH, salinity, and organic matter can affect their persistence and antibacterial activity. Consequently, phage-based interventions should be evaluated not only under laboratory conditions but also under real-world environmental settings to accurately assess their effectiveness and practical feasibility.

## 4 Applications of bacteriophages in medicine, veterinary science, and environmental settings

### 4.1 Historical background and clinical renaissance

The therapeutic application of bacteriophages was pioneered by Félix d’Hérelle in 1917, who first demonstrated their antibacterial potential against dysentery and cholera. Following the introduction of antibiotics in the 1940s, phage therapy was progressively abandoned in most Western countries. In contrast, it remained in continuous clinical use in Eastern Europe, particularly at the Eliava Institute (Tbilisi, Georgia) and the Hirszfeld Institute (Wrocław, Poland), where thousands of patients have been treated over several decades (Górski et al., 2020).

The current global burden of multidrug-resistant (MDR) bacteria has renewed scientific and clinical interest in bacteriophages. This resurgence is reflected in an increasing number of compassionate use applications and early-phase clinical investigations, positioning phage therapy as a promising adjunct or alternative to conventional antimicrobials in selected refractory infections.

### 4.2 Clinical applications in human medicine

#### 4.2.1 Compassionate use cases

Modern clinical evidence for phage therapy is largely derived from compassionate use cases involving life-threatening MDR infections. A landmark example is the treatment of disseminated, pan-resistant *Acinetobacter baumannii* infection in a patient (2016), where a personalized intravenous phage cocktail, developed through a collaboration between the University of California San Diego and the U.S. Navy Medical Research Center, contributed to full clinical recovery (Schooley et al., 2017). This case established an important precedent for systemic phage administration in the United States.

Similarly, in France, clinical teams at Lyon University Hospital have reported successful outcomes using locally administered phage preparations in combination with surgical debridement and antibiotics for prosthetic joint infections caused by *Staphylococcus aureus* and *Pseudomonas aeruginosa* (Ferry et al., 2020). These experiences supported the implementation of regulated compassionate access pathways through the French National Agency for the Safety of Medicines and Health Products.

Another pivotal case involved a pediatric lung transplant recipient with disseminated *Mycobacterium abscessus* infection refractory to all available antibiotics. Treatment with a three-phage cocktail, including a genetically engineered phage to broaden host range, resulted in significant clinical and microbiological improvement over a prolonged treatment period (Dedrick et al., 2019). This case expanded the therapeutic scope of phage therapy to genetically modified phage applications and highly recalcitrant intracellular pathogens.

#### 4.2.2 Clinical trials

Beyond individual cases, controlled clinical evidence remains limited but is steadily growing. The PhagoBurn trial (France, Belgium, Switzerland), the first randomized controlled trial of topical phage therapy in burn patients infected with *Pseudomonas aeruginosa*, confirmed the overall safety of phage preparations but highlighted critical formulation and manufacturing challenges, including insufficient phage titers at the point of care (Jault et al., 2019). These findings underscored the importance of standardized GMP-compliant production pipelines.

Ongoing and planned trials, including PHAGECYA in France and Phase I/II studies in Europe, the United States, and Australia, are evaluating phage therapy against MDR pathogens such as *Escherichia coli*, *Klebsiella pneumoniae*, MRSA, and *Clostridioides difficile* (Pirnay et al., 2024). Collectively, these studies aim to generate robust clinical evidence regarding efficacy, dosing strategies, and delivery routes.

#### 4.2.3 Regulatory frameworks

Despite increasing clinical interest, the regulatory status of phage therapy remains heterogeneous and fragmented. In Europe, no unified regulatory framework currently exists for bacteriophage medicinal products. Belgium has implemented a magistral preparation system allowing hospital pharmacies to formulate phages under prescription. France permits compassionate use via temporary authorization mechanisms (ATU/early access schemes), while Georgia and Poland operate under long-standing national clinical frameworks (Pirnay et al., 2018).

In the United States, phage therapy is regulated through the FDA Investigational New Drug (IND) pathway, including expanded access programs for individual patients. However, the lack of harmonized international standards for production, quality control, and clinical trial design, particularly given the self-replicating nature of phages, remains a major barrier to widespread clinical translation.

The fragmentation of regulatory processes also has specific scientific implications, as it dissuades multinational research designs and makes direct comparison of efficacy more difficult, while making patient access to treatment dependent on geographical factors rather than on medical ones. The lack of a coordinated regulatory process (similar to that in place for conventional antimicrobial drugs) may result in the continued fragmentation of the scientific evidence base into small jurisdictional studies rather than convergence to larger randomized trials.

### 4.3 Applications in veterinary medicine and animal husbandry

#### 4.3.1 Livestock

Given the role of agricultural antibiotic use in driving antimicrobial resistance (Section 2.2.2), bacteriophages have been extensively explored as alternatives to prophylactic and growth-promoting antibiotics in food-producing animals. In poultry, phage cocktails targeting *Salmonella enteritidis* and *Salmonella typhimurium* administered via drinking water have been shown to reduce intestinal colonization, thereby improving both animal health and food safety (Hagens and Loessner, 2010).

In experimentally infected broilers, Loc Carrillo et al. (2005) reported reductions of up to 2 log₁₀ CFU/g in *Campylobacter jejuni caecal* loads following oral phage administration, highlighting the potential of phages to reduce a major foodborne pathogen associated with extensive antibiotic use.

In swine production, phages targeting enterotoxigenic *Escherichia coli* (ETEC), a primary cause of post-weaning diarrhea, have demonstrated reductions in bacterial shedding and disease severity, supporting their potential to reduce antibiotic dependency during critical production phases (Jamalludeen et al., 2009).

#### 4.3.2 Aquaculture

Aquaculture represents a high-risk environment for antimicrobial resistance emergence due to intensive antibiotic use. Phage therapy has therefore been evaluated against major aquatic pathogens including *Aeromonas hydrophila*, *Vibrio harveyi*, and *Flavobacterium columnare*. Experimental studies in fish and shrimp systems have demonstrated reduced mortality and pathogen burden following phage treatment, without detectable adverse effects on host physiology or microbiota composition (Nakai and Park, 2002).

Given the limited availability of approved veterinary antibiotics in aquaculture, phage-based strategies represent a particularly relevant and sustainable alternative for disease control in this sector.

### 4.4 Approved commercial products

Several bacteriophage-based products have received regulatory authorization for food safety and agricultural applications, demonstrating the translational potential of phage biocontrol in non-clinical settings. Table 2 provides a comprehensive overview of currently approved phage-based commercial products.

**Table 2:** Summary of commercially approved bacteriophage-based products.

Product	Developer	Target organism	Application sector	Regulatory status/region	References
ListShield™	Intralytix (USA)	*Listeria monocytogenes*	ready-to-eat meat and poultry products	FDA GRAS; USA	Intralytix, 2006; FDA GRAS Notice GRN No. 000198
EcoShield PX™	Intralytix (USA)	*Escherichia coli* O157: H7	Beef products	FDA food safety clearance; USA	Intralytix, 2011; FDA Food Contact Substance Notification (FCN)
SalmoFresh™	Micreos (Netherlands)	*Salmonella spp*	food processing environments	FDA authorised; Netherlands	Intralytix, 2013; FDA GRAS Notice GRN No. 000435
Agriphage®	OmniLytics (USA)	*Xanthomonas campestris* and *Pseudomonas syringae*	Crop protection (tomato, pepper)	EPA-registered biopesticide; USA	OmniLytics, 2002; U. S. EPA Registration No. 67986–1
INTESTI phage	Eliava Institute (Georgia)	Enteric pathogens	Clinical (enteric infections)	National clinical approval; Georgia	no publicly available FDA/EPA/EMA registration or patent identified
Staphylococcal phage Sb-1	Eliava Institute (Georgia)	*Staphylococcus aureus* (incl. MRSA)	Clinical (wound/ENT infections)	National clinical approval; Georgia, Poland	no publicly available FDA/EPA/EMA registration or patent identified

These regulatory approvals highlight that bacteriophage preparations can meet stringent safety and efficacy requirements in food and agricultural systems, thereby establishing a validated framework for their potential expansion into broader environmental and clinical applications.

### 4.5 Quantitative evidence for environmental phage applications

A growing number of research articles offer quantifiable data on environmental phage intervention, highlighting both its advantages and limitations, thus complementing mechanisms described above.

Phage cocktails aimed at MDR *P. aeruginosa* proved effective in decreasing both planktonic cell numbers and biomass of biofilm in biofilms. Synergy effect of phage action with sub-inhibitory antibiotic doses was also revealed (Chegini et al., 2020). Results of this study are especially interesting for discussion of phages in WWTP, because biofilm communities attached to reactor surfaces are the sources of persistent reservoirs of resistant bacteria and mobile resistance elements.

Phage cocktails were found to be highly effective in reducing *Klebsiella pneumoniae, Enterococcus faecium,* and *Acinetobacter baumannii* in conditions of real hospital wastewater, resulting in eradication of *K. pneumoniae* over a six-month monitoring period. Nonetheless, regrowth of bacteria was observed during following months, possibly due to selection of resistant phage variants, which indicates the need for adaptive strategies in phage treatment (Sadeqi et al., 2022). Limitations of efficiency of phage intervention in wastewater are often observed in studies of different phages; thus, there is an obvious necessity to conduct more extensive monitoring of long-term results of phage action before large-scale applications.

In food and meat matrices, lytic phages targeting *E. coli* O157: H7 applied directly to beef surfaces achieved reductions of 0.77–1.15 log₁₀ CFU/g within 6 h at temperatures ranging from 5 to 24 °C, with the emergence of phage-insensitive mutants noted as a recurring limitation (Tomat et al., 2013).

As was mentioned in Section 4.3, application of phages for intervention in aquaculture and food production facilities demonstrated quite effective results, with several preparations receiving commercial regulatory approval.

These studies prove efficiency of phage-based interventions as a perspective supplementary strategy in environmental AMR control. At the same time, effectiveness of this strategy varies widely according to matrix and conditions, stability of phages and emergence of resistance remain main problems for broad deployment in environmental settings. Summary of results of phage-based interventions is presented in Table 3.

**Table 3:** Summary of reported phage-based interventions in environmental matrices against antibiotic-resistant bacteria.

Matrix	Target bacteria	Phage type	Conditions	Key outcomes	Limitations	References
Wastewater biofilm	*P. aeruginosa* MDR	Lytic cocktail	Lab-scale reactor	Significant reductions in planktonic counts and biofilm biomass; synergy with antibiotics	Phage-resistant mutants; matrix-dependent efficacy	Chegini et al. (2020)
Hospital wastewater	KPC-producing *Klebsiella pneumoniae*	Lytic cocktail	Real hospital effluent	Significant reduction of nosocomial pathogens	Bacterial regrowth due to phage-resistant variants	Sadeqi et al. (2022)
Meat surfaces	*E. coli* O157: H7	Lytic phages (DT1, DT6)	Beef, 5–24 °C, 6 h	0.77–1.15 log₁₀ CFU/g reduction	Emergence of phage-insensitive mutants	Tomat et al. (2013)
Livestock / poultry	*Salmonella Typhimurium*	SalmoFresh™ (commercial)	Field application	Significant reduction on carcasses and surfaces	regulatory constraints on open-field use	Hagens and Loessner (2010)
Aquaculture	*Vibrio* spp.	Lytic phage preparations	Semi-field	Reduced Vibrio counts; decreased antibiotic use	Temperature and salinity sensitivity	Nakai and Park (2002)
Aquaculture	*Aeromonas salmonicida*	Lytic phages (feed/water)	Lab and semi-field	Reduced furunculosis mortality	Variable efficacy by water temperature	Nakai and Park (2002)
Food processing	*E. coli* O157: H7	EcoShield PX™ (commercial)	Field application	Significant reduction on meat surfaces	Limited to food-contact surfaces	Hagens and Loessner (2010)

## 5 Emerging phage-based technologies

Bacteriophages have not only been used as therapeutic and bio-control agents but have become a driving force behind many technological innovations in the field of biosensors, food safety, and environmental remediation by taking advantage of their specificity and adaptability.

### 5.1 Phage-based biosensors for pathogen and ARG detection

There is an increasing trend towards utilization of phages and phage protein molecules, e.g., receptor binding proteins, as bioreceptor components in biosensor devices that use electrochemical, optical, or acoustic transduction. They allow the rapid and sensitive identification of pathogenic microorganisms such as *E. coli*, *Salmonella*, and *S. aureus* at concentrations of ~10^2^ CFU/mL within 30–60 min, surpassing the culture techniques (Al-Hindi et al., 2022).

In a more recent application, the technology of phage biosensors was adopted for the screening of resistant bacteria in the environment (Magnano San Lio et al., 2023).

### 5.2 Bioactive packaging and disinfectant incorporating bacteriophages for food processing and environmental settings

The integration of bacteriophages in food packaging materials, ranging from biopolymer films, edible coatings, and electrospun nanofibers, has gained recognition as a potentially viable approach to combat foodborne bacteria and increase the shelf life of food items. The effectiveness of phage-packaging combination has been shown against various foodborne bacteria, including *Listeria monocytogenes, Salmonella, and Escherichia coli* O157: H7 strains, resulting in reduction of bacterial load. Ensuring stability of phages in packaging materials is among the major difficulties associated with integration of bacteriophages in packaging materials. However, using encapsulation technology (alginate and PEG based systems) can increase the viability and infectivity of the phages. The growing regulatory acceptance of phage products due to their GRAS status (ListShield™ and EcoShield PX™), which are commercially available, stimulates development of phage-packaging materials for organic foods with fewer preservatives (Lone et al., 2016; Vonasek et al., 2018). In parallel, phages are being designed to act as liquid decontaminants and surface disinfectants in the food processing industry as well as in clinical settings. Their high specificity makes them ideal for removing pathogenic microorganisms without damaging equipment surfaces or generating toxic residues (Chen et al., 2024).

### 5.3 Genetically engineered and synthetic phages

Development of techniques of synthetic biology and CRISPR-Cas technology of genome editing has made possible rational engineering of bacteriophages having advanced therapeutic features. There are three major approaches: increasing the spectrum of hosts by means of tail fibre or receptor-binding protein modification; creating obligately lytic mutants of temperate bacteriophages by removing sequences responsible for lysogenicity; and equipping bacteriophages with CRISPR-Cas elements that specifically target certain ARGs and virulence genes, thus providing selective elimination of resistant bacterial strains from the complex population of microorganisms (Kiga et al., 2020). The first clinical experiment conducted with the use of engineered therapeutic phage that successfully treated disseminated infection caused by *Mycobacterium abscessus* resistant to all existing antibiotics confirmed the applicability of this technology (Dedrick et al., 2019). However, despite obvious potential of genetic engineering of phages in case of precise AMR control, regulatory issues of their usage are poorly developed, and strict ecological risk assessment should be conducted prior to any environmental applications.

## 6 Constraints, limitations, strategies to overcome them, and future perspectives

### 6.1 Bacterial resistance to phages and strategies to overcome it

Bacteria have evolved several antiphage defense systems such as receptor modification or deletion, restriction-modification (R-M) systems, abortive infection (Abi) systems, and CRISPR-Cas adaptive immune system. Resistance mutants to phages could be generated per generation depending on the selective pressure. To address this challenge, various approaches have been developed to overcome this problem (Ibrahim and Aranjani, 2026).

First, the use of mixed phages which infect bacteria through different receptors reduces greatly the risk of the generation of resistant mutants and is now commonly used in phage therapy (Kunz Coyne et al., 2024). In addition, phage-antibiotic synergy (PAS) uses sub-inhibitory concentrations of antibiotics to induce phage receptor expression, disruption of biofilm and resistance mutation formation (Kunz Coyne et al., 2024; Tchatchiashvili et al., 2026). Furthermore, directed evolution methods by serial passage on resistant strains could efficiently increase the host range of phages (Kunz Coyne et al., 2024). Finally, more recent molecular approaches, including the use of anti-CRISPR proteins and CRISPR-Cas-based phage engineering, have been developed to circumvent bacterial defense mechanisms and to generate broad-host-range phages, respectively (Duan et al., 2024).

These strategies do not eliminate the risk of phage resistance but rather aim to reduce or delay its emergence, reflecting the continuous evolutionary arms race between bacteria and phages. The use of phage combinations and phage–antibiotic synergy therefore requires continuous monitoring and periodic adaptation to bacterial evolution, raising questions about the long-term feasibility and cost-effectiveness of maintaining and updating phage libraries.

### 6.2 Narrow host range and environmental stability

Despite its positive implications for specific biocontrol applications, the high specificity of phages towards their hosts restricts their potential use in diverse environments where many bacteria coexist at the same time. Characterization of the bacteria population prior to applying phages requires the use of metagenomics, but may be impractical in case of urgent situations. In addition, survival of the phages in the environment depends on various factors such as temperature, pH level, UV exposure, ionic strength, and proteases activity. Development of phage formulations (microencapsulation, lyophilisation, and inclusion of protective agents) is currently a popular area of research (Malik et al., 2023).

Other than formulation stability, the ecological impacts associated with phage introduction at a large scale in the environment have not been thoroughly evaluated. The introduction of high amounts of lytic phages to complex microbiomes can have unanticipated effects on non-target bacteria, change the composition of the microbiome, and select for phage-resistant strains with changes in virulence/fitness (Muneeb et al., 2025). From laboratory/pilot to operational scale, such as million-liter-per-day WWTPs, also poses practical obstacles that hinder field implementation (Reyneke et al., 2024).

### 6.3 Risk of phage-mediated gene transfer and biosafety considerations

The ability of some phages to facilitate horizontal gene transfer via transduction gives rise to significant biosafety concerns with respect to their intended environmental release. Complete genomic analysis of the phages in question is thus a necessity before their application to ensure that these do not contain ARGs, virulence factors, integrases or any homologies to known MGEs (Ansaldi et al., 2020). Only strictly lytic phages with well-characterized genomes should be considered for environmental application. More generally, the regulatory approaches concerning the environmental use of phages are still immature in comparison to scientific advances. In the US, agricultural bacteriophage biopesticides are regulated by the EPA under FIFRA legislation, whereas food contact phages are regulated by the FDA using the GRAS approach. In the EU, the classification of products based on phages, namely whether they constitute medicinal products, plant protection agents, or novel foods, is currently debated among regulators (Yang et al., 2023).

In addition to classification under regulatory frameworks, the development of environmental risk assessment (ERA) frameworks for bacteriophages is less advanced when compared to the frameworks that exist for chemical substances and genetically modified organisms (Gulig et al., 2024). There are several critical gaps that have yet to be addressed, including lack of protocols for measuring transduction rates in the field, lack of defined risk levels that are deemed acceptable in terms of the mobilization of ARGs, and lack of long-term data on the survival and horizontal gene transfer of phages after their environmental release. Similar problems were raised by the European Commission’s Joint Research Centre in their 2024 report on this topic, whereas the OECD has released guidance documents specifically for phage evaluation as plant protection products (Baud et al., 2026). Development of harmonized ERA guidelines, perhaps borrowing from the ERA frameworks already in place for biopesticides or probiotics, is one of the priorities.

### 6.4 Future directions and research priorities

Achieving advancements towards large-scale application of phages into the environment and clinical setting would demand progress along several related axes. In particular, future research conducted under the One Health paradigm should integrate surveillance of the environmental resistome with the selection and optimization of the appropriate phage cocktails for the identified bacterial populations from environmental hotspots. There is a great need for pilot-scale environmental investigations of the effect of the phage-based interventions under operational conditions in WWTPs, in agriculture, and aquaculture. Longitudinal monitoring of the effect of the use of phages on the overall composition of the environmental resistome is critical as well. It would be essential to perform systematic studies of HGT mediated by phages in realistic environmental conditions in order to provide quantifiable risk data for regulatory decision making. Finally, development of rapid and deployable metagenomic surveillance tools, together with standardized libraries of phage cocktails against important pathogenic and resistant bacteria in the environment would provide the way to develop an intelligent approach to phage application.

## 7 Conclusion

The environmental component of AMR has increasingly been appreciated as one of the major factors associated with the global AMR threat since the environment acts as an important source of antibiotic-resistant bacteria and ARGs due to sewage, health care waste water, agricultural practices, and landfills. Although there have been substantial advancements in surveillance technologies for detecting ARGs in the environment, the problem with their practical application remains open.

In this respect, bacteriophages are considered a novel and mechanistically unique approach for dealing with antibiotic-resistant bacteria not only in clinical practice but also in veterinary and environmental settings because of their lytic properties coupled with the activities of phage enzymes and anti-biofilm properties that allow targeting the elimination of multidrug-resistant pathogens. Phages have been successfully used in the development of innovative technologies ranging from biosensing to active food packaging as well as phage-surface and phage-food treatments.

There is increasing proof of the applicability of the technology both in terms of treating serious infections and regulatory approvals of phage products used in food biocontrol. The phage-based strategies have demonstrated effectiveness also in wastewater treatment, health care waste water treatment, and food processing settings.

However, despite its advantages, this technology has some limitations associated with the narrow host range, emergence of phage-resistant variants, stability of phages in the environment, and horizontal gene transfer risk caused by bacteriophages. Various techniques such as phage cocktails, phage-antibiotics combinations, directed evolution, and genetic modification are being explored to cope with these difficulties. In addition, there are some major gaps in the existing literature: lack of quantitative data on transduction rates from natural environments, lack of standard procedures for screening phages’ safety before their release into the environment, and lack of information regarding the long-term impact of mass deployment of phages on the structure of microbial communities. Thus, ultimately, bacteriophages should be considered as a part of comprehensive One Health approach involving surveillance, wastewater treatment, antibiotic stewardship, and ecological risk assessment of phages.
