# Supplementary material for: Bacteriophages in the environmental control of antimicrobial resistance: opportunities, risks, and surveillance challenges
Source: Front Microbiol. 2026 Sep 25;17:1938962. doi: 10.3389/fmicb.2026.1938962 (PMC13650914; doi:10.3389/fmicb.2026.1938962)
Supplement: Supplementary file 1 [file Table_1.docx]

**Supplementary Table 1: ARGs Wastewater Treatment Plants**

| S/N | Country (Location) | Source | Time of the year | Genes | Method of Isolation | Findings | Source |
| --- | --- | --- | --- | --- | --- | --- | --- |
| 1 | ShenZhen, China | FuTian full-scale wastewater treatment plant |  | *aac(2)-I, aac(6")-1, aac(6')-II, aadA, ant(2"")-I aph(3")-I, aph(6)-I, bacA, AER-1* class A,  *СТХ-M-*group2, *GES-*group1,  *IMP*-group2, *ОХА-1/4/47,*  *OXA-12, OXA-5/129, OXA-3,*  *OXA-46/205, OXA-*group2  *OXA-*group3, *PSE-1,* *TEM*-group1*, VEB-*group1, *catB, cMIA, floR, rOSA, rosB, ksgA, ereA, ermB, ermc, ermE,*  *macA, macB, mphA, acrB, adeB, amrB, emrA, emrB, emrD, emrE, mdfA, mdtA, mdtB, mdtC, mdtE, mdtK, mdtL, mexB, mexC, mexD, mexE, mexF, mexT, mexW,*  multidrug_ABC, multidrug_transporter, *отр36*, *ompR, gacEdelta1, arnA, gnrB,*  *9nrS, arr, sull, sul2, sul4, tet39, tetA, tetC, tetG, tetQ, tetv, tetX2, dfrA1/A5, dfrA12, dfrA14, dfrA15, dfrA16, dfrA7/A17, dfrB6, Camp, cob(I), H-NS, CpxR, vanR* | Metagenomic sequencing (ARGPore2) illumina | The findings showed 33 genera of antibiotic-resistant bacteria carrying 65 ARG subtypes in the receiving seawater, with concentration 10 times higher than that of clean seawaters. | (Wu et al. 2022) |
| 2 | Changzhou, China | Samples from different treatment units of a WWTP, receiving river water, lake water, tap water, and human feces. |  | *strB* and *bla_TEM-1_* were detected in all water samples | qPCR | These findings suggest a potential ARG interchange between human feces and receiving water bodies. | (Chen et al. 2023) |
| 3 | China | WWTPs receiving  domestic sewage, including health infrastructure sewage |  | *tetM, tetO, tetQ, tetW, sul1, sul2* | qPCR | No dissimilarity was found among the three municipal WWTPs with different treatment processes | (Chen and Zhang, 2013) |
| 4 | Hongkong, China | Sewage treatment plant | July, August, September, November and December of 2011 and January | *tet32*  *tetA*  *tetA(33)*  *tetA(M)*  *tetA(Q)*  *tetB(P)*  *tetG*  *tetM*  *tetO*  *tetQ*  *tetW*  *tetX*  beta-lactamase  *NPS-1*  *OXA* gene  *OXA-46*  penicillin binding protein  penicillin-binding protein 2  *VEB-1*  *VEB-3*  *VIM-11* | Metagenomics sequencing (Illumina) | The concentrations of ARGs and antibiotic-resistant bacteria in all effluent samples was lower than that in the influent samples, which revealed that the WWTPs can effectively remove majority of ARGs and antibiotic-resistant bacteria in pig farm wastewater. | (Yang et al., 2020) |
| 5 | Tanzania | wastewater | - | *Sul1, Sul2, Tet A, Tet B-1, Tet D, bla_CTX-M_, bla _SHV_* | PCR amplification | Ten isolates contained the tetracycline-resistant genes in which 4 isolates contained the Tet A, Tet B - 1 and 5 isolates contained Tet D. Moreover β-lactamases (*bla_CTX-M_ and bla_SHV_*) were detected in 7 isolates. | (Ripanda et al., 2023) |
| 6 | Germany | WWTP | February till November, 2017 | *sul1 intl1 bla_TEM_ ermB tetM CTX-M-32 bla_OXA_-_48_ CTX-M bla_VIM_ CMY-2 vanA mcr-1 bla_NDM_* | qPCR | The results revealed that both ultrafiltration and ozonation could decrease the concentration majority of ARGs (e.g. *mcr-1, bla_NDM-1_, vanA, and bla_VIM_*) and pathogenic bacteria | (Chen and Zhang, 2013) |
| 7 | Portugal | urban household, hospital and a wastewater treatment plant | April and July | *bla_TEM_, bla_OXA_, bla_CTX-M-1_, bla_CTX-M-9_, bla_EBC_, bla_FOX_,*  *bla_DHA_, bla_CIT_* | PCR amplification | The findings showed that the largest contribution to the spread of multidrug resistant bacteria is found to be hospital and the WWTP. | (Amador et al., 2015) |
| 8 | China | drinking water treatment plants |  | *sul 1*  *sul 2*  *sul 3*  *tetA*  *tetB/P*  *tetC*  *tetH*  *tetM*  *tetO*  *tetQ*  *tetS*  *tetT*  *tetW*  *tetX*  *ermB*  *ermC*  *cmlA*  *floR*  *fexA*  *fexB*  *cfr* | qPCR | It was found that *Pseudomonas* could be contributing to the proliferation of ARGs in the drinking water treatment system. | (Su et al., 2018) |
| 9 | Canada | waste water | September and November 2015 | *bla_CTX-M_, int1, bla_TEM_, sul1, sul2, mecA, tetO, qnrS, ermB* | qPCR | Although wastewater treatment reduced the overall abundance of ARGs, these genes remained detectable in the effluent. The presence of *Pseudomonas* spp. suggests that certain bacterial populations may contribute to the persistence and potential proliferation of ARGs within water treatment systems. | (Neudorf et al., 2017) |
| 10 | USA | Wastewater treatment plants | May and July | *bla_TEM_, intl1, tetW, qnrA, qnrB, qnrS, sul1, ermB* | Metagenomics sequencing (Illumina) | Secondary treatment increased the diversity of potential ARG hosts, while the addition of chlorination broadened the host range of certain resistance genes, indicating that treatment processes can influence the dissemination of ARGs among different bacterial populations. | (Cheng et al., 2021) |
| 11. | USA | Raw water, finished water from a water treatment plant, and tap water | - | *sul1* and *tet(A)* (present in raw water but absent in other sources) | PCR | The water treatment plant successfully removed the antibiotic-resistant bacteria in the treated water as none of the bacteria were detected in the tap water. | (Bergeron et al., 2015) |
| 12 | Canada | two activated sludge treatment plants and two physicochemical treatment plants | - | *aac (3)-IIa (aacC2)*  *ant (3")-Ia (aadA1)*  *ant (2")-Ia (aadB)*  *aph (3')-Ia (aphA1)*  *aadA7*  *aph3 (strA)*  *aph6 (strB)*  *aphA-3*  *ereA*  *ereB*  *mphA*  *ereA2*  *bla_OXA-1_*  *bla_OXA-7_*  *bla_PSE-4_*  *bla_TEM_*  *ampR (bla_DHA-1_* repressor)  *bla_CTX_M12_*  *bla__SME1_*  *bla__VIM2_*  *qnrS1*  *qnrS2*  *catIII*  *cat*  *catB2*  *cat*  *sulII*  *sulIII*  *sulI*  *tet(A)*  *tet(B)*  *tet(E)*  *tet(M)*  *tet(Q)*  *tet(30)*  *tetX*  *tet(G)*  *dhfrI*  *dhfrXV*  *dhfrV*  *dhfrVII*  *dhfrIX*  *dfrA20*  *dhfrVIII*  *dhfrXII*  *qac*  *oqxA*  *oqxB* | DNA microarray | The results showed that treatment of activated sludge did not modify the occurrence of ARG-possessing. | (Biswal et al., 2014) |
| 13 | Hong Kong, China | wastewater treatment plants (WWTPs) in | - | *aac(6)-I*  *aadA*  *aadA2*  *aadA3*  *ANT (2*)-la*  *aph(6)-Id* Aminoglycoside  *AAC (6)-Ib-cr*  *bla_A_*  *NPS-1*  *OXA-11*  *VEB-3*  *VEB-7*  *caB*  *cmIA*  *floR*  *ereA -*  *mefC*  *mphD*  *mphG*  *qurs*  *sull*  *sul2*  *tet(39)*  *tet(C)*  *tet(G)*  *tet(A)*  *tet(E)*  *dfrA14.*  *CfxA*  *mef(A/E)*  *tetM*  *tetQ*  *OXA-347*  *MOX-6*  *VEB-9*  *ermF*  *InuB*  *macB*  *tet(E)*  *tet(X)*  *tetw* | metagenomics sequencing on the Illumina | The results showed that mobile genetic elements-associated ARGs dominated the resistome in WWTPs and their concentration increased in the effluent. | (Che et al., 2019) |
| 14 | China | Two Effluent receiving areas of WWTP | April of 2017 and 2018 | *tetA*  *tetC*  *tetO*  *tetW*  *sull*  *sullI*  *dfrAl*  *dfrA13*  *bla_pse-1_*  *ermB*  *floR*  *intIl* | qPCR using SYBR Green I approach | Sul genes were the richest ARG subtype in the two ERAs, followed by tet genes in these two years, while the concentration of ARGs in 2018 was found to be higher than that in 2017. | (Chen et al., 2020) |
| 15 | China | WWTP | April | *aadA6*  *aadA1*  *aac(6’)-lla*  *aac(6’)-lb3*  *ant(2”)-la*  *aac(6’)-lb-cr*  *erm(F)*  *floR*  *catB4*  *sul2*  *sul1* | metagenomics sequencing on the Illumina | The results showed that sewage sludge produced by this WWTP are treated through incineration and dewatering which helped to prevent the spread of antibiotic-resistant genes and ARGs. | (Jiang et al., 2019) |
| 16 | Poland | river water and wastewater effluents from WWTPs | November 2017 (autumn), 6–7 February 2018 (winter), 16–17 April 2018 (spring) and 16–17 July 2018 (summer). | beta-lactams (*bla_TEM_*), tetracyclines (*tet(A)*), macrolides, lincosamides and streptogramins (*ermF*), sulfonamides (*sul1*), and fluoroquinolones *(aac(6′)-Ib-cr)* | qPCR | The highest concentrations of the ARGs were observed in the effluent samples from small WWTPs. In addition, concentration of ARGs reached a peak in winter and spring seasons | (Harnisz et al., 2020) |
| 17 | South Africa | 5 WWTPs that receive domestic sewage, hospital and farmland wastewaters | From August 2015 to December 2015 | *Asa, Esp, lsa, emeA, erfB, erfA, aac(6‘)-Ie-aph(2“)-Ia, aph(3‘)-IIIa, vanC, tetL, tetM, ermB* | qPCR | The presence of enterococci harbouring ARGs in the treated wastewater propose that ARGs are discharged into the environment where their spread could be extended. | (Hamiwe et al., 2019; Scott et al., 2021) |
| 18 | USA | Soil and water from Rocky Mountain National Park | June  2018 to October 2018 | *TetA*  *TetD*  *gnIB*  *gnIS*  *VanA* | qPCR | Antibiotic-resistant bacteria and ARGs were detected in soil and water samples collected from sites with low human impact in Rocky Mountain National Park. | (Ekwanzala et al., 2020; Jankowski et al., 2022) |
| 19 | Agadir, Morocco | 6 WWTPs | February and July 2020 | 223 ARGs spanning 31 antibiotic classes (aminoglycosides, MLS, β-lactams, sulfonamides, tetracyclines, chloramphenicol) | Metagenomic sequencing  (NGS) | Despite treatment, many ARGs remained detectable in effluents. Co-occurrence with high levels of mobile genetic elements indicates potential horizontal gene transfer and environmental dissemination | (Wardi et al., 2024b) |

| S/N | Country (Location) | Source | Time of the year  **Supplementary Table 2: ARGs in Farms** | Genes | Method of Isolation | Findings | Source |
| --- | --- | --- | --- | --- | --- | --- | --- |
| 1. | northern Italy | organic broiler farm and slaughter house | January | *bla_TEM_,*  *qnrS,*  *sul2,*  *tetA* | qPCR | The concentration of the ARGs detected in farm environment was generally comparable to that measured in non-antibiotic free farms. This indicates that chicks could be colonized by antibiotic-resistant bacteria at their arrival. | (Salerno et al., 2022) |
| 2. | Sichuan Province of China | duck slaughterhouse | May 2016 to July 2017 | virulence determinants in Salmonella isolates:  *avrA,*  *ssaQ,*  *mgtC,*  *siiD,*  *sopB,*  *bcfC,*  *spvR,*  *spvB,*  *spvC* | PCR | Salmonella isolates showed multidrug resistance and carried multiple virulence-associated genes which possess health risks to consumers. | (Han et al., 2020) |
| 3. | KwaZulu-Natal, South Africa | At each production stage of poultry from farm to fork (including farm and slaughterhouse) | August and September | *tetM,*  *ermB,*  *aph(3′)-IIIa,*  *vanC1* | PCR | The results show the high incidence of MDR Enterococcus isolates containing both resistance and virulence genes in diverse forms which can serve as a reservoir for the potential transfer of these genes from poultry to humans | (Molechan et al., 2019) |
| 4. | Amman City, Jordan | Calves slaughtered at Amman slaughter | January to May | Virulent genes in *E.coli* detected  (*eaeA, hlyA, stx1, stx2*) | PCR | Fifty *E.coli* O157:H7 isolates were identified and virulence factors *eaeA* and *hlyA* were present in all of the isolates. | (Osaili et al., 2013) |
| 5. | China | Cattle, poultry and swine farms |  | *tetW, tetO, tetM, sul1, sul2, qepA, qnrB, qnrS, ermB, and ermF* | PCR | Poultry and swine manure have higher concentration of ARGs than cow manures. | (Wang et al., 2013) |
| 6. | Ghana | Water samples and fish samples |  | *bla_TEM_*  *bla_TEM-1_*  *bla_EBC_*  *bla_CTX-M_*  *bla_mecA_*  *Sul1*  *Sul3*  *Cat1*  *cmIA*  *gyrA*  *qnrB*  *qnrS*  *tetB* | PCR | The majority of bacterial isolates showed a high resistance to beta-lactam antibiotics. | (Agbeko et al., 2022) |
| 7. | Long Province, Vietnam | water and sediment samples from shrimp farm effluent channels |  | *sul1,*  *sul2,*  *qnrA,*  *tetA,*  *aac(6′)-lb-cr,*  *ermB,*  *dfrA1,*  *dfrA12,*  *dfrA5* | PCR | These findings showed the presence of very high concentration of the ARG *sul2* in the effluent of a shrimp farming area, as well as in the receiving water body. | (Pham et al., 2018) |
| 8. | Italy | Marine bacteria from aquaculture farms |  | *bla_AmpC_*  *bla_OXA-55-like_*  *bla_CARB-19_* | PCR | Aquatic environment plays a role as a reservoir for antimicrobial resistance gene. | (Zago et al., 2020) |
| 9. | Beijing, China | Air, feces, and feed samples from a Poultry farm without antibiotics |  | *strA*  *strB*  *qnrS*  *qnrA*  *ermA*  *ermB*  *ermC*  *sul1*  *sul2*  *sul3*  *tetM*  *tetG*  *tetC*  *tetO* | qPCR | The concentration of airborne ARGs with in-feed antibiotics is higher than that with no drugs. | (Song et al., 2022) |
| 10. | Jeju Island, South Korea | Fish farm effluent and non-fish farm seawater were collected | Summer and winter | *aph3a*  *CARB-3*  *mefC*  *pp-flo*  *QnrS2*  *sul2*  *tet(59)*  *tet(B)* | qPCR | The results indicated an increased concentration of ARGs, virulence factors, and pathogens in the fish farm effluents when compared to non-fish farm seawater. | (Jo et al., 2021) |
| 11. | Shandong, China | wastewater samples swine farms | September to November | *aadA,*  *aacC2,*  *aphA3,*  *aacC4,*  *bla_CTX-M_,*  *bla_TEM_,*  *bla_SHV_,*  *bla_OXA_,*  *tetA,*  *tetB,*  *sul1,*  *sul2,*  *sul3,*  *qnrA,*  *qnrB,*  *qnrS* | PCR | The E. coli isolated from swine-farm wastewater were mainly resistant to tetracyclines (95.3%), chloramphenicol (77.8%), and sulfonamides (62.2%), while antibiotic resistance genes for quinolones, tetracyclines, sulfonamides, aminoglycosides, and β-lactams were all more than 60% | (Liu et al., 2022) |
| 12. | Guangzhou, China | Treated wastewater samples from  pig farms, layer farms, and broiler farms |  | *sul1, sul2, strB, ermA, ermB, tetA, tetW, tetX, bla_TEM_, mcr-1, NDM-1, intl1* and *tnpA* | qPCR | The results showed that the treated wastewater from livestock farms was an important reservoir for ARGs, and the concentration of ARGs in broiler farms was higher than that in other types of livestock farms | (Chen et al., 2023) |
| 13. | Heilongjiang Province, China | Soil samples from dairy farmland | June | *sul2, tetX, bla_TEM_, tetAP , sul1, tetM, tetW, cfxA , tetPA, tetZ, tetG , sul3 , tetT, tetS , bla_OXA1_/bla_OXA30_ , tetY , tetC , tetO , tetB , fox5 , tet36 , tet34, bla_PSE_ ,tet32, ampC2 , tetR, bla_OXA10_ , ampC4* | PCR | The concentration of β-lactam ARGs (*β-RGs*) increased with TCs and β-Ls increased. The concentration of tetracycline ARGs (*TRGs*) remained relatively stable with increasing concentrations of all antibiotics, while that of sulfonamide ARGs (*SRGs*) tend to decrease. | (Qi et al., 2022) |
| 14. | Southern Thailand | Rectal swabs from goats | January - July | *aadA1, aadA2, bla_TEM-1B_, cmlA1, dfrA12, floR, qnrS1, sul1, sul2, sul3, tet(A)* | whole-genome sequencing analysis | The findings showed that implementation of antibiotic resistance surveillance programs in goat production is important | (Pomwised et al., 2023) |
| 15. | Guangdong Province, China | manure sample from workers, pigs, environmental samples and vegetable sampes in the swine farm |  | ARGs shared by swine, workers and environment:  *AAC (3)-IId, aadA2, aadS, ANT (6)-Ib, APH (3'')-Ib, APH (3')-IIIa, APH (6)-Id, OXA-347, dfrA17, lnuC, EreD, ErmF, mel, sul2, SAT-4, floR, tet(40), tet(A), tetA(P), tetM, tetW* | Metagenomic sequencing | The study revealed a high concentration of ARGs in manure samples (both swine and workers) compared to environmental samples on the farm. | (Tong et al., 2022) |
| 16. | Guangdong, China | Samples of Duck feces, water, sediment, and shrimp were collected from a duck–shrimp integrated farm |  | *TetM* was the most dominant ARG subtype, followed by ARG subtypes *vanS, ant (9)-I*, and *bacA.* | Metagenomic sequencing | The results indicate that duck feces, with the most diverse and abundant ARGs. | (Zhu et al., 2021) |
| 17. | Guangdong Province, southern China | Samples of airborne bacteria, animal feces, and dust were collected from a chicken farm and a dairy farm |  | *sul1, tetG, tetW, cfr, cmlA, fexA, aadA, qnrS, mecA, intl1, intl2, Tn916, floR* | PCR | Higher concentration and diversity of  airborne antibiotic-resistant bacteria were detected in air of the poultry farm than in the dairy farm. | (Bai et al., 2022) |
| 18. | Guangdong Province, China | Manure, layer manure fertilizer and soil samples from laying hen farms |  | *tetA, tetM, tetQ, tetX, ermB, ermC, sul1, sul2, bla_TEM_, oqxB, qnrS, cfr,*  *strB, int1* | PCR | There was a higher concentration of ARGs in poultry manure during the incubation period than in other periods. | (Zhu et al., 2021) |
| 19. | China | Water samples from pig farms and fish ponds |  | *aac (6')-Ib(aka aacA4)*  *aadA*  *aadA1*  *aadA2*  *aadA5*  *aadE*  *bla_CTX-M_*  *bla_GES_*  *cmlA1*  *ereA*  *ermF*  *acrA*  *emrD*  *qacEdelta1*  *qacH*  *dfrA1*  *sul2*  *tetG*  *tetM*  *tetPB*  *tetX*  *intI-1(clinic)*  *tnpA-04*  *tnpA-05*  *tnpA-07* | HT-qPCR | Both the pig farm and fish ponds were vital hotspots for ARGs in this area, and the pig farm was found to have more ARGs and higher diversity of microbiota than fish ponds | (Fu et al., 2022) |
| 20. | Italy | pooled faecal samples and boot swab from cattle farm |  | *bla_TEM_/bla_SHV_/bla_OXA-1-like_* genes and *bla_CTX-M-1/2/9_* genes. | PCR | This study has showed that cow-calf beef farms are a potential source of AROs and ARGs for humans. | (Ferroni et al., 2022) |
| 21 | Guangdong Province, China | Pig farm waste water treatment plant | May | *bla_kPc_*  *bla_NDM_*  *cmlA*  *qnrs*  *strB*  *cfr*  *oqхВ*  *ermA*  *ermB*  *sul1*  *sul2*  *tetA*  *fetC*  *tetG*  *tetH*  *tetL*  *tetM*  *tetO*  *tetQ*  *tetw*  *tetX* | qPCR | This study revealed an increased concentration of ARGs in pig farm WWTPs. | (Yang et al., 2020) |

| S/N  **Supplementary Table 3: ARGs in Hospitals** | Country (Location) | source | Time of the year | Genes | Method of Isolation | Findings | Source |
| --- | --- | --- | --- | --- | --- | --- | --- |
| 1 | Poland | Hospital, municipal sewage, and wastewater | - | *bla_CTX-M_, bla_TEM_, bla_OXA_* and *bla_SHV_* | Metagenomic sequencing (fluorescent DNA sequencer) | The preliminary disinfection of hospital sewage before their inflow into the sewage system could reduce the dispersion of antibiotic-resistant bacteria to environment | (Korzeniewska et al., 2013) |
| 2 | Germany | clinical wastewater,  municipal WWTP  Groundwater,  Rain overflow basins,    Surface water | - | *bla_VIM-1_*  *vanA*  *ampC*  *ermB* | qPCR | Increased population of opportunistic bacteria and ARG were identified in clinical wastewaters and influents of the adjacent WWTP. | (Alexander et al., 2015) |
| 3 | Japan | hospital effluent, sewage treatment plant (STP) and river water |  | *CRE*  *ESBL*  *MDRA*  *MDRP*  *MRSA*  *VRE*  *Escherichia coli*  *Coliform* group | chromogenic agar media | These findings show the importance of decreasing the concentrations of environmental pollutants in sewage treatment plant before the discharge of effluent into the surface waters. | (Azuma et al., 2019) |
| 4 | Spain | hospital and WWTP effluents | November, December and January | *bla_TEM_,*  *qnrS,*  *ermB,*  *sulI and*  *tetW* | qPCR | The results also revealed an increased concentrations of ARGs, such as *bla_TEM_, qnrS, ermB, sulI* and *tetW* in hospital effluent and WWTP influent samples. | (Rodriguez-Mozaz et al., 2015) |
| 5 | Belgium | treated sewage waters from WWTP and sewage waters from hospital | - | *bla_NDM_, bla_KPC_, bla_OXA-48_* | qPCR | An increased concentration of ARGs were recorded in hospital waters. Although absolute abundances were decreased in WWTP effluents, the relative abundance was never lowered through wastewater treatment | (Proia et al., 2018) |
| 6 | DR Congo | Water and sediments samples were collected from untreated effluents at a hospital and a clinic | March/April 2019 (rainy season) and in June/August, 2019 (dry season) | *bla_OXA-48_, bla_CTX-M,_ bla_IMP_,*  *bla_TEM_* | qPCR | The results show that hospitals contribute significantly in both seasons total and antibiotic-resistant E. coli and Enterobacteriaceae to the sediment, which acts as reservoir for these bacteria. | (Al Salah et al., 2020) |
| 7 | China | Aerosol samples collected around a general tertiary hospital, adjacent urban and sub-urban areas | January, April, July, and October in 2017 | *fmtC*  *GES-13*  *PBP-1A*  *PBP-1B*  *PBP-2X*  *PER-1*  *PER-7*  *Erm (36)*  *lnuA*  *mphC*  *msrA*  *vgaA*  *EmrB-QacA* family major facilitator transporter  *mepA*  *mtrE*  *norA*  *qacA*  *qacB*  *qnrS*  *tetB*  *aac(3)-IV*  *aad(6)*  *aadE*  *ant(9)-I*  *aph(2'')-II*  *aph(3''')-III*  streptomycin resistance protein  chloramphenicol and florfenicol resistance gene  *ermB*  *vatE*  *tet44*  *tetL*  *tetM*  *tetO*  *tetP*  *tetQ*  *tetW*  *tetZ*  *dfrA17* | Metagenomics (SARG: Structured Antibiotic Resistance Genes database) | The results of this study suggest a prevalence of airborne ARG contamination in urban hospitals, nearby urban communities and the nearest suburban communities. The chloramphenicol exporter gene, *sul1, bacA* and *lnuA* were the most abundant ARG subtypes among the air samples. | (He et al., 2020a) |
| 8 | China | Hospital wastewater influent and effluent samples from 3 hospitals | September 2018 to July 2019 in four seasons (i.e., spring, summer, autumn and winter) | Vancomycin,  Trimethoprim,  Tetracenomycin_C,  Sulfonamide,  Rifamycin,  Quinolone,  Puromycin,  Polymyxin,  Multidrug,  MLS,  Kasugamycin,  Fosfomycin,  Chloramphenicol,  Bleomycin,  Beta-lactam,  Bacitracin,  aminoglycoside | Metagenomics (SARG database) | The findings highlighted the risk of hospital wastewater based on the diversity, abundance, mobility, and host pathogenicity of ARGs. | (Zhu et al., 2023) |
| 9. | China | Wastewater and sludge samples from hospital | November to December 2020 | aminoglycoside *(aadA6, aac(6′)-Ib),* beta-lactams *(bla_VEB_, bla_MOX_/bla_CMY_, and bla_NDM_),* MLSB *(ere(A), ere(B), msr(E)),* multidrug *(qacH_351),* sulfonamide *(sul1)* resistance genes*, and* MGE encoding genes *(IS21-ISAs29, IS6100,* and *intI1).* | HT-qPCR | An increased prevalence of ARGs and bacterial pathogens from the feces of patients was recorded in hospital Wastewater Treatment Plant. | (Bian et al., 2023) |
| 10. | Cape Town, South Africa | Samples of Bacteroides fragilis strains isolated from microbiology laboratory | - | *cfiA* gene | PCR | The results showed that none of the metronidazole resistant strains carried the *nimA-J* genes*,* and no *cfxA* or *ermF* genes were detected. | (Meggersee and Abratt, 2015) |

| S/N  **Supplementary Table 4: ARGs in Landfills** | Country (Location) | Source | Time of the year | Genes | Method of Isolation | Findings | Source |
| --- | --- | --- | --- | --- | --- | --- | --- |
| 1 | China | Landfills | spring, summer, fall, and winter | *tetO* (2.03 × 10^−7^ to 4.26 × 10^−2^),  *tetW* (2.03 × 10^−7^ to 4.06 × 10^−3^)  *sulI* (5.51 × 10^−6^ to 4.97 × 10^−1^)  copy of ARG/copy of 16S rRNA gene | Metagenomics sequencing (Illumina) | These results revealed that antibiotic resistance in landfill and landfill leachate have high health risk and the kind of ARGs with high abundance in human-associated environment, gene mobility and pathogenicity should be paid more attention | (Wang et al., 2022) |
| 2 | China | waste landfill | August 2020 to September 2020 | *msrE,*  *sul1,*  *sul2,*  *aa dA* | ddPCR (Bio-Rad) | Results revealed that aerosols released during waste recycling had a significant impact on composition of environmental microorganisms and cause the spread of ARGs. | (Tao et al., 2022) |
| 3. | China | Landfill leachates samples from fresh (0–1-yr-old), middle-aged (3–5-yr-old), and aged (5–10-yr-old) landfills. | Rainy season (June) and the dry season (December) | The order of abundance of ARGs is *mexF > sul2 > Intl1 > sul1 > ermB > mefA > tetM > tetQ.* | qPCR | The total concentration of antibiotics decreased with increasing leachate age.  The profile of antibiotics and ARGs in leachates was affected by several factors, and the pollution of antibiotics and ARGs may be decreased and controlled by adjusting the environmental factors. | (Liu et al., 2022) |
| 4. | Nanjing, China | Soils were sampled bi-monthly from 3 landfill locations | February 2014 to February 2015 | two quinolone resistance genes *(qnrA* and *qnrS),* two β-lactams resistance genes *(ampC* and *bla_SHV_),* two macrolides resistance genes *(ermA* and *ermB),* two sulfonamide resistance genes *(sulI and sulII*), five tetracycline resistance genes *(tetM, tetW, tetB, tetG,* and *tetX),* and two chloramphenicol resistance genes *(cmlA* and *catI)* | qPCR | Mass migration of people, the quantity of dumped waste and temperature have the highest effects on bimonthly fluctuations of ARG levels in landfill sites. | (Sun et al., 2016) |
| 5. | Xiamen, China | Landfills Leachate samples, influent (LInf) and effluent (LEff) of the leachate treatment plant |  | A total of 203 unique ARGs and 10 MGEs were detected in all leachate and groundwater samples. Eight categories (aminoglycoside, beta-lactams, chloramphenicol, macrolide lincosamide streptogramin B resistance (MLSB), multidrug, sulfonamide, tetracycline and vancomycin) of ARGs based on the type of antibiotics, were classified in samples | HT-qPCR | The number of detected ARGs and their abundance in groundwater increased as the distance from the leachate basin decreased, suggesting a potential leakage of leachate into groundwater and spread of ARGs in the corresponding hydrogeological groundwater unit. | (Huang et al., 2022) |
| 6. | China | Bioaerosol samples from landfill | September, 2020 | *tnpA03*  *IS1111*  *intl1*  *bla_OXA10_*  *bla_SHV_*  *bacA*  *mdtF*  *mexF*  *acrF*  *acrB*  *sul2*  *sul1*  *tetX*  *tetM*  *tetC*  *tetA*  *ermB*  *aadA* | qPCR | Pathogenic bacteria (Bacilli, Bacillus, and Burkholderia-Paraburkholderia) and allergenic fungi (Aspergillus, Cladosporium, and Curvularia) prevailed in landfill. | (Liang et al., 2023) |
| 7. | Shanghai, China | Leachates samples from six landfill columns |  | *bla_TEM_, bla_OXA_, strB,* *aadA1,* *ermB, mefA, sul1, sul2, tetM, tetQ* | qPCR | Target ARGs were found to associate with pathogens in leachates. | (Wu et al., 2021) |
| 8. | China | Refuse samples were collected with mechanical equipment in triplicate at every 2.0 m |  | *sul1, sul2, tetM, tetQ, ermB, mefA, mexF, int1* | qPCR | The concentration of antibiotics and the abundance of genes varied with increasing depth of landfill. | (Li et al., 2022) |
| 9. | Beijing, China | Refuse samples colleted from 2 areas in the ladfills: an inactive zone (no longer receiving waste with an average deposit age > 10 years) and an active landfill unit (with an average deposit age < 3 years) |  | A total of 154 ARG subtypes were detected and among them, the *ermT-01*, *tetX*, *sul2*, *aadA-02* and *aadA2–03* were identified as the core ARGs.  A set of 67 ARGs were shared by all of the samples and their sum abundance accounted for 93.1–99.7% of the total ARGs abundance. Among these shared ARGs, the *ermT-01, tetX, sul2, aadA-02* and *aadA2–03* were found to be the five most abundant ARGs | qPCR | The total abundance of ARGs (ARG transcripts) in young refuse was higher than that in old refuse | (Xu et al., 2021) |
| 10. | China | Refuse samples were collected from 10 small- and medium-sized landfills | July to November 2018 | *qnrD, qnrS, mexF,* *ermA, ermB, mefA,* *sul1* and *sul2*  fluoroquinolone-related ARGs (*qnrD, qnrS*, and *mexF*), macrolide-related ARGs (*ermA, ermB*, and *mefA*), and sulfonamide-related ARGs (*sul1* and *sul2*). | qPCR | The relative abundances of *qnrD, qnrS, mexF, ermA, ermB, mefA, sul1,* and *sul2* in most landfills were >10−4 copies per 16S rRNA, suggesting the presence of highly contaminated ARGs | (Yao et al., 2020) |
| 11. | China | Refuse samples were collected from thirteen small- or medium-sized landfills |  | *tetM, tetQ, tetW, tetX, ampC, bla_CTX-M_, bla_PER_, bla_SFO_, bla_SHV_, emrA, emrB, mefA, qnrD, qnrS, mexF, sul1, sul2.* | qPCR | Abundance of *tetM, tetX, bla_PER_, emrB, sul1* and *sul2* increased with refuse age. | (Chen et al., 2021) |
| 12. | Italy | Landfill leachate samples | October in 2016 to February in 2017 | *IntI, sulI,sulII, ermB, tetW, tetM, tetA, tetQ, mefA, cat.* | qPCR | 9 ARGs and 1 MGE were all detected in leachate spanning 34 years.  Aging leachate and landfill mining have potential risk of antibiotic resistance. | (He et al., 2021) |
| 13 | China | influent (INF) and effluent (EFF) from the landfill leachate waste treatment station, as well as samples of river water were collected |  | A total of 91 ARGs 5 MGEs were detected, and the ARGs encode resistance to antibiotics that have been extensively administered to humans, which can be divided into the major classes such as:  Aminoglycoside  Beta_Lactams  Chloramphenicol  Integron  MLSB  Multidrug  Sulfonamide  Tetracycline  Transposase  Vancomycin  Others | qPCR | This study showed the risk of discharging processed landfill leachate in spread of antibiotic resistance determinants to the environments. | (Wang et al., 2020) |

**References**

Agbeko R, Aheto DW, Asante DKA, Asare NK, Boateng AA and Adinortey CA (2022). Identification of molecular determinants of antibiotic resistance in some fish farms of Ghana. Heliyon 8, e10431. doi: 10.1016/j.heliyon.2022.e10431.

Al Salah DMM, Ngweme GN, Laffite A, Otamonga J-P, Mulaji C and Poté J (2020). Hospital wastewaters: A reservoir and source of clinically relevant bacteria and antibiotic resistant genes dissemination in urban river under tropical conditions. Ecotoxicol. Environ. Saf. 200, 110767. doi: 10.1016/j.ecoenv.2020.110767.

Alexander J, Bollmann A, Seitz W and Schwartz T (2015). Microbiological characterization of aquatic microbiomes targeting taxonomical marker genes and antibiotic resistance genes of opportunistic bacteria. Sci. Total Environ. 512–513, 316–325. doi: 10.1016/j.scitotenv.2015.01.046.

Amador PP, Fernandes RM, Prudêncio MC, Barreto MP and Duarte IM (2015). Antibiotic resistance in wastewater: Occurrence and fate of Enterobacteriaceae producers of Class A and Class C β-lactamases. J. Environ. Sci. Health A 50, 26–39. doi: 10.1080/10934529.2015.964602.

Azuma T, Otomo K, Kunitou M, Shimizu M, Hosomaru K, Mikata S, et al. (2019). Environmental fate of pharmaceutical compounds and antimicrobial-resistant bacteria in hospital effluents, and contributions to pollutant loads in the surface waters in Japan. Sci. Total Environ. 657, 476–484. doi: 10.1016/j.scitotenv.2018.11.433.

Bai H, He L-Y, Wu D-L, Gao F-Z, Zhang M, Zou H-Y, et al. (2022). Spread of airborne antibiotic resistance from animal farms to the environment: Dispersal pattern and exposure risk. Environ. Int. 158, 106927. doi: 10.1016/j.envint.2021.106927.

Bergeron S, Boopathy R, Nathaniel R, Corbin A and LaFleur G (2015). Presence of antibiotic resistant bacteria and antibiotic resistance genes in raw source water and treated drinking water. Int. Biodeterior. Biodegradation 102, 370–374. doi: 10.1016/j.ibiod.2015.04.017.

Bian J, Wang H, Ding H, Song Y, Zhang X, Tang X, et al. (2023). Unveiling the dynamics of antibiotic resistome, bacterial communities, and metals from the feces of patients in a typical hospital wastewater treatment system. Sci. Total Environ. 858, 159907. doi: 10.1016/j.scitotenv.2022.159907.

Biswal BK, Mazza A, Masson L, Gehr R and Frigon D (2014). Impact of wastewater treatment processes on antimicrobial resistance genes and their co-occurrence with virulence genes in Escherichia coli. Water Res. 50, 245–253. doi: 10.1016/j.watres.2013.11.047.

Che Y, Xia Y, Liu L, Li A-D, Yang Y and Zhang T (2019). Mobile antibiotic resistome in wastewater treatment plants revealed by Nanopore metagenomic sequencing. Microbiome 7, 44. doi: 10.1186/s40168-019-0663-0.

Chen H and Zhang M (2013). Effects of advanced treatment systems on the removal of antibiotic resistance genes in wastewater treatment plants from Hangzhou, China. Environ. Sci. Technol. 47, 8157–8163. doi: 10.1021/es401091y.

Chen J, Zhang Z, Lei Z, Shimizu K, Yao P, Su Z, et al. (2020). Occurrence and distribution of antibiotic resistance genes in the coastal sediments of effluent-receiving areas of WWTPs, China. Bioresour. Technol. Rep. 11, 100511. doi: 10.1016/j.biteb.2020.100511.

Chen Z, Duan Y, Yin L, Chen Y, Xue Y, Wang X, et al. (2023). Unraveling the influence of human fecal pollution on antibiotic resistance gene levels in different receiving water bodies using crAssphage indicator gene. J. Hazard. Mater. 442, 130005. doi: 10.1016/j.jhazmat.2022.130005.

Chen Z, Yao L, Sun F, Zhu Y, Li N, Shen D, et al. (2021). Antibiotic resistance genes are enriched with prolonged age of refuse in small and medium-sized landfill systems. Environ. Res. 197, 111194. doi: 10.1016/j.envres.2021.111194.

Cheng X, Xu J, Smith G and Zhang Y (2021). Metagenomic insights into dissemination of antibiotic resistance across bacterial genera in wastewater treatment. Chemosphere 271, 129563. doi: 10.1016/j.chemosphere.2021.129563.

Ekwanzala MD, Dewar JB and Momba MNB (2020). Environmental resistome risks of wastewaters and aquatic environments deciphered by shotgun metagenomic assembly. Ecotoxicol. Environ. Saf. 197, 110612. doi: 10.1016/j.ecoenv.2020.110612.

Ferroni L, Albini E, Lovito C, Blasi F, Maresca C, Massacci FR, et al. (2022). Antibiotic consumption is a major driver of antibiotic resistance in calves raised on Italian cow-calf beef farms. Res. Vet. Sci. 145, 71–81. doi: 10.1016/j.rvsc.2022.01.010.

Fu C, Ding H, Zhang Q, Song Y, Wei Y, Wang Y, et al. (2022). Comparative analysis of antibiotic resistance genes on a pig farm and its neighboring fish ponds in a lakeside district. Environ. Pollut. 303, 119180. doi: 10.1016/j.envpol.2022.119180.

Hamiwe T, Kock MM, Magwira CA, Antiabong JF and Ehlers MM (2019). Occurrence of enterococci harbouring clinically important antibiotic resistance genes in the aquatic environment in Gauteng, South Africa. Environ. Pollut. 245, 1041–1049. doi: 10.1016/j.envpol.2018.11.040.

Han X, Peng J, Guan X, Li J, Huang X, Liu S, et al. (2020). Genetic and antimicrobial resistance profiles of Salmonella spp. isolated from ducks along the slaughter line in southwestern China. Food Control 107, 106805. doi: 10.1016/j.foodcont.2019.106805.

Harnisz M, Kiedrzyńska E, Kiedrzyński M, Korzeniewska E, Czatzkowska M, Koniuszewska I, et al. (2020). The impact of WWTP size and sampling season on the prevalence of antibiotic resistance genes in wastewater and the river system. Sci. Total Environ. 741, 140466. doi: 10.1016/j.scitotenv.2020.140466.

He P, Huang J, Yu Z, Xu X, Raga R and Lü F (2021). Antibiotic resistance contamination in four Italian municipal solid waste landfills sites spanning 34 years. Chemosphere 266, 129182. doi: 10.1016/j.chemosphere.2020.129182.

He P, Wu Y, Huang W, Wu X, Lv J, Liu P, et al. (2020). Characteristics of and variation in airborne ARGs among urban hospitals and adjacent urban and suburban communities: A metagenomic approach. Environ. Int. 139, 105625. doi: 10.1016/j.envint.2020.105625.

Huang F-Y, Zhou S-Y-D, Zhao Y, Zhou X-Y, Li H, Zhang X, et al. (2022). Dissemination of antibiotic resistance genes from landfill leachate to groundwater. J. Hazard. Mater. 440, 129763. doi: 10.1016/j.jhazmat.2022.129763.

Jankowski P, Gan J, Le T, McKennitt M, Garcia A, Yanaç K, et al. (2022). Metagenomic community composition and resistome analysis in a full-scale cold climate wastewater treatment plant. Environ. Microbiome 17, 3. doi: 10.1186/s40793-022-00398-1.

Jiang X, Cui X, Xu H, Liu W, Tao F, Shao T, et al. (2019). Whole Genome Sequencing of Extended-Spectrum Beta-Lactamase (ESBL)-Producing Escherichia coli Isolated From a Wastewater Treatment Plant in China. Front. Microbiol. 10, 1797. doi: 10.3389/fmicb.2019.01797.

Jo H, Raza S, Farooq A, Kim J and Unno T (2021). Fish farm effluents as a source of antibiotic resistance gene dissemination on Jeju Island, South Korea. Environ. Pollut. 276, 116764. doi: 10.1016/j.envpol.2021.116764.

Korzeniewska E, Korzeniewska A and Harnisz M (2013). Antibiotic resistant Escherichia coli in hospital and municipal sewage and their emission to the environment. Ecotoxicol. Environ. Saf. 91, 96–102. doi: 10.1016/j.ecoenv.2013.01.014.

Li H, Qiu L, Chen B, Wang H, Liu H, Long Y, et al. (2022). Vertical distribution of antibiotics and antibiotic resistance genes in a representative municipal solid waste landfill, China. Ecotoxicol. Environ. Saf. 242, 113919. doi: 10.1016/j.ecoenv.2022.113919.

Liang Z, Yu Y, Wang X, Liao W, Li G and An T (2023). The exposure risks associated with pathogens and antibiotic resistance genes in bioaerosol from municipal landfill and surrounding area. J. Environ. Sci. 129, 90–103. doi: 10.1016/j.jes.2022.09.038.

Liu H, Li H, Qiu L, Chen B, Wang H, Fang C, et al. (2022). The panorama of antibiotics and the related antibiotic resistance genes (ARGs) in landfill leachate. Waste Manag. 144, 19–28. doi: 10.1016/j.wasman.2022.03.008.

Meggersee R and Abratt V (2015). The occurrence of antibiotic resistance genes in drug resistant Bacteroides fragilis isolates from Groote Schuur Hospital, South Africa. Anaerobe 32, 1–6. doi: 10.1016/j.anaerobe.2014.11.003.

Molechan C, Amoako DG, Abia ALK, Somboro AM, Bester LA and Essack SY (2019). Molecular epidemiology of antibiotic-resistant Enterococcus spp. from the farm-to-fork continuum in intensive poultry production in KwaZulu-Natal, South Africa. Sci. Total Environ. 692, 868–878. doi: 10.1016/j.scitotenv.2019.07.324.

Osaili TM, Alaboudi AR and Rahahlah M (2013). Prevalence and antimicrobial susceptibility of Escherichia coli O157:H7 on beef cattle slaughtered in Amman abattoir. Meat Sci. 93, 463–468. doi: 10.1016/j.meatsci.2012.11.023.

Pham TTH, Rossi P, Dinh HDK, Pham NTA, Tran PA, Ho TTKM, et al. (2018). Analysis of antibiotic multi-resistant bacteria and resistance genes in the effluent of an intensive shrimp farm (Long An, Vietnam). J. Environ. Manage. 214, 149–156. doi: 10.1016/j.jenvman.2018.02.089.

Proia L, Anzil A, Borrego C, Farrè M, Llorca M, Sanchis J, et al. (2018). Occurrence and persistence of carbapenemases genes in hospital and wastewater treatment plants and propagation in the receiving river. J. Hazard. Mater. 358, 33–43. doi: 10.1016/j.jhazmat.2018.06.058.

Qi Z, Le Z, Han F, Qi Y and Liu R (2022). β-lactamase genes transmission influenced by tetracycline, sulfonamide and β-lactams antibiotics contamination in the on-site farm soil. Ecotoxicol. Environ. Saf. 241, 113753. doi: 10.1016/j.ecoenv.2022.113753.

Ripanda AS, Rwiza MJ, Nyanza EC, Miraji H, Bih NL, Mzula A, et al. (2023). Antibiotic-resistant microbial populations in urban receiving waters and wastewaters from Tanzania. Environ. Chem. Ecotoxicol. 5, 1–8. doi: 10.1016/j.enceco.2022.10.003.

Rodriguez-Mozaz S, Chamorro S, Marti E, Huerta B, Gros M, Sànchez-Melsió A, et al. (2015). Occurrence of antibiotics and antibiotic resistance genes in hospital and urban wastewaters and their impact on the receiving river. Water Res. 69, 234–242. doi: 10.1016/j.watres.2014.11.021.

Salerno B, Furlan M, Sabatino R, Di Cesare A, Leati M, Volanti M, et al. (2022). Antibiotic resistance genes load in an antibiotic free organic broiler farm. Poult. Sci. 101, 101675. doi: 10.1016/j.psj.2021.101675.

Scott LC, Wilson MJ, Esser SM, Lee NL, Wheeler ME, Aubee A, et al. (2021). Assessing visitor use impact on antibiotic resistant bacteria and antibiotic resistance genes in soil and water environments of Rocky Mountain National Park. Sci. Total Environ. 785, 147122. doi: 10.1016/j.scitotenv.2021.147122.

Song L, Jiang G, Wang C, Ma J and Chen H (2022). Effects of antibiotics consumption on the behavior of airborne antibiotic resistance genes in chicken farms. J. Hazard. Mater. 437, 129288. doi: 10.1016/j.jhazmat.2022.129288.

Su H-C, Liu Y-S, Pan C-G, Chen J, He L-Y and Ying G-G (2018). Persistence of antibiotic resistance genes and bacterial community changes in drinking water treatment system: From drinking water source to tap water. Sci. Total Environ. 616–617, 453–461. doi: 10.1016/j.scitotenv.2017.10.318.

Sun M, Ye M, Schwab AP, Li X, Wan J, Wei Z, et al. (2016). Human migration activities drive the fluctuation of ARGs: Case study of landfills in Nanjing, eastern China. J. Hazard. Mater. 315, 93–101. doi: 10.1016/j.jhazmat.2016.04.077.

Tao J, Chen Y, Wan S and Wu J (2022). Abundance and diversity of ARGs in aerosol environments of waste recycling sites. J. Aerosol Sci. 165, 106020. doi: 10.1016/j.jaerosci.2022.106020.

Tong C, Xiao D, Xie L, Yang J, Zhao R, Hao J, et al. (2022). Swine manure facilitates the spread of antibiotic resistome including tigecycline-resistant tet(X) variants to farm workers and receiving environment. Sci. Total Environ. 808, 152157. doi: 10.1016/j.scitotenv.2021.152157.

Wang J-Y, An X-L, Huang F-Y and Su J-Q (2020). Antibiotic resistome in a landfill leachate treatment plant and effluent-receiving river. Chemosphere 242, 125207. doi: 10.1016/j.chemosphere.2019.125207.

Wang Y, Zhang R, Lei Y and Song L (2022). Antibiotic resistance genes in landfill leachates from seven municipal solid waste landfills: Seasonal variations, hosts, and risk assessment. Sci. Total Environ. 853, 158677. doi: 10.1016/j.scitotenv.2022.158677.

Wang Z, Zhang XX, Huang K, Miao Y, Shi P, Liu B, et al. (2013). Metagenomic profiling of antibiotic resistance genes and mobile genetic elements in a tannery wastewater treatment plant. PLoS One 8, e76079. doi: 10.1371/journal.pone.0076079.

Wardi M, Lemkhente Z, Ait Alla A, Slimani N, Abali M, Idaghdour Y and Belmouden A (2024). Resistome analysis of wastewater treatment plants in Agadir city, Morocco, using a metagenomics approach. Sci. Rep. 14, 26328. doi: 10.1038/s41598-024-76773-4.

Wu D, Wang L, Su Y, Dolfing J and Xie B (2021). Associations between human bacterial pathogens and ARGs are magnified in leachates as landfill ages. Chemosphere 264, 128446. doi: 10.1016/j.chemosphere.2020.128446.

Wu Z, Che Y, Dang C, Zhang M, Zhang X, Sun Y, et al. (2022). Nanopore-based long-read metagenomics uncover the resistome intrusion by antibiotic resistant bacteria from treated wastewater in receiving water body. Water Res. 226, 119282. doi: 10.1016/j.watres.2022.119282.

Xu S, Qasim MZ, Zhang T, Wang R, Li C and Ge S (2021). Diversity, abundance and expression of the antibiotic resistance genes in a Chinese landfill: Effect of deposit age. J. Hazard. Mater. 417, 126027. doi: 10.1016/j.jhazmat.2021.126027.

Yang Y, Chen Y, Cai Y, Xing S, Mi J and Liao X (2020). The relationship between culturable doxycycline-resistant bacterial communities and antibiotic resistance gene hosts in pig farm wastewater treatment plants. Ecotoxicol. Environ. Saf. 206, 111164. doi: 10.1016/j.ecoenv.2020.111164.

Yao L, Li Y, Li Z, Shen D, Feng H, Zhou H, et al. (2020). Prevalence of fluoroquinolone, macrolide and sulfonamide-related resistance genes in landfills from East China, mainly driven by MGEs. Ecotoxicol. Environ. Saf. 190, 110131. doi: 10.1016/j.ecoenv.2019.110131.

Zago V, Veschetti L, Patuzzo C, Malerba G and Lleo MM (2020). Shewanella algae and Vibrio spp. strains isolated in Italian aquaculture farms are reservoirs of antibiotic resistant genes that might constitute a risk for human health. Mar. Pollut. Bull. 154, 111057. doi: 10.1016/j.marpolbul.2020.111057.

Zhu L, Yuan L, Shuai X-Y, Lin Z-J, Sun Y-J, Zhou Z-C, et al. (2023). Deciphering basic and key traits of antibiotic resistome in influent and effluent of hospital wastewater treatment systems. Water Res. 231, 119614. doi: 10.1016/j.watres.2023.119614.

Zhu T, Chen T, Cao Z, Zhong S, Wen X, Mi J, et al. (2021). Antibiotic resistance genes in layer farms and their correlation with environmental samples. Poult. Sci. 100, 101485. doi: 10.1016/j.psj.2021.101485.
